# Wg/Wnt1 and Erasp link ER stress to proapoptotic signaling in an autosomal dominant retinitis pigmentosa model

**DOI:** 10.1038/s12276-023-01044-7

**Published:** 2023-07-18

**Authors:** Jung-Eun Park, Jiyoun Lee, Soonhyuck Ok, Seunghee Byun, Eun-Ju Chang, Sung-Eun Yoon, Young-Joon Kim, Min-Ji Kang

**Affiliations:** 1 Department of Pharmacology, Asan Medical Center, University of Ulsan College of Medicine, Seoul, 05505 Republic of Korea; 2 School of Biopharmaceutical and Medical Sciences, Sungshin University, Seoul, 01133 Republic of Korea; 3 Department of Biochemistry and Molecular Biology, Asan Medical Center, University of Ulsan College of Medicine, Seoul, 05505 Republic of Korea; 4 Biomedical Research Center, Asan Institute for Life Sciences, Asan Medical Center, 88, Olympic-ro 43-gil, Songpa-gu, Seoul, 05505 Republic of Korea; 5 Korea Drosophila Resource Center, Gwangju Institute of Science and Technology (GIST), 123 Cheomdangwagi-ro, Buk-gu, Gwangju, 61005 Republic of Korea; 6 School of Life Sciences, Gwangju Institute of Science and Technology (GIST), 123 Cheomdangwagi-ro, Buk-gu, Gwangju, 61005 Republic of Korea

**Keywords:** Apoptosis, Endoplasmic reticulum

## Abstract

The endoplasmic reticulum (ER) is a subcellular organelle essential for cellular homeostasis. Perturbation of ER functions due to various conditions can induce apoptosis. Chronic ER stress has been implicated in a wide range of diseases, including autosomal dominant retinitis pigmentosa (ADRP), which is characterized by age-dependent retinal degeneration caused by mutant rhodopsin alleles. However, the signaling pathways that mediate apoptosis in response to ER stress remain poorly understood. In this study, we performed an unbiased in vivo RNAi screen with a *Drosophila* ADRP model and found that Wg/Wnt1 mediated apoptosis. Subsequent transcriptome analysis revealed that ER stress-associated serine protease (Erasp), which has been predicted to show serine-type endopeptidase activity, was a downstream target of Wg/Wnt1 during ER stress. Furthermore, knocking down *Erasp* via RNAi suppressed apoptosis induced by mutant rhodopsin-1 (Rh-1^P37H^) toxicity, alleviating retinal degeneration in the *Drosophila* ADRP model. In contrast, overexpression of Erasp resulted in enhanced caspase activity in *Drosophila* S2 cells treated with apoptotic inducers and the stabilization of the initiator caspase Dronc (Death regulator Nedd2-like caspase) by stimulating DIAP1 (*Drosophila* inhibitor of apoptosis protein 1) degradation. These findings helped identify a novel cell death signaling pathway involved in retinal degeneration in an autosomal dominant retinitis pigmentosa model.

## Eye disease: a pathway to cell death in retinitis pigmentosa

A *Drosophila* fly model of an inherited form of the eye disease retinitis pigmentosa has revealed a signaling and control pathway involving the intracellular endoplasmic reticulum structure that leads to cell death. Retinitis pigmentosa is an age-related disease of the retina of the eye, leading to progressive vision loss. It is known to involve dysfunction of the endoplasmic reticulum, which, among other functions crucial for cell health, is essential for the synthesis of many proteins. Researchers in South Korea led by Min-Ji Kang at the University of Ulsan College of Medicine identified specific genes and proteins involved in their *Drosophila* model of RP, which may also be significant in human disease. These insights may contribute to preventing the progression of retinitis pigmentosa in human patients.

## Introduction

Signaling pathways related to stress responses help organisms survive under hostile conditions, either by protecting cells through damage repair and adaptation or by eliminating damaged cells through active cell death programs, such as apoptosis. The induction of cell death is beneficial for tissues in which lost cells can be readily replaced. In contrast, certain conditions that trigger the death of irreplaceable vital cells, including ER stress-induced cell death, underlie numerous neurodegenerative, digestive, and metabolic diseases in humans and model organisms^1^.

Stress responses of the endoplasmic reticulum (ER), triggered by the accumulation of unfolded proteins in the ER lumen, lead to the activation of multiple intracellular signaling pathways; this response is widely referred to as the unfolded protein response (UPR)^2^. Among known UPR pathways, a pathway regulated by *IRE1* and *XBP1* has been extensively characterized. IRE1 is a transmembrane ER protein with a luminal domain that can sense the presence of misfolded proteins^3,4^. The endonuclease domain in the cytoplasmic region of IRE1 catalyzes *XBP1* mRNA splicing. The resultant frameshift in XBP1 mRNA translation generates an active isoform of XBP1^5–7^. In addition to the IRE1/XBP1 pathway, two other well-characterized UPR pathways are regulated by ATF6 and PERK/ATF4. The critical functions of these pathways lead to the ultimate restoration of ER capacity by inducing the transcription of genes that encode ER chaperones, antioxidant proteins, and proteins involved in the degradation of misfolded proteins^2^.

Similar to cells under other stress conditions, certain cells are vulnerable to apoptosis under conditions of chronic ER stress. Consistently, the expression of a number of pro-apoptotic genes, including *Bim, Noxa*, and *FasR*, is induced in response to ER stress^8–10^, suggesting the activation of signaling pathways that promote cell death. Given that a better understanding of such pro-apoptotic pathways may pave the way for developing new approaches for treating ER stress-associated degenerative disease, models that can be used to elucidate the relevant pathways have been proposed. These models include a pro-apoptotic branch of signaling that originates from the IRE1 or ATF4 pathways^11,12^, proteolytic activation of caspases in response to abnormal Ca^2+^ levels^13,14^, and pathways that are initiated by excessive reactive oxygen species (ROS) that are generated in the stressed ER^15–17^. However, these models have no consensus, and their pathological significance still needs to be clarified.

The Wnt signaling pathway is a crucial regulator of embryonic development across species and governs homeostatic self-renewal in diverse adult tissues^18–20^. In addition to its roles in cell proliferation and differentiation during development, Wnt signaling regulates retinal development through apoptosis. Inactivation of a *Drosophila* homolog of the tumor suppressor adenomatous polyposis coli (APC) or Armadillo overexpression, which mediates canonical Wnt signaling, causes retinal degeneration via apoptosis. This phenotype is similar to that observed in humans with congenital hypertrophy of the retinal pigment epithelium (CHRPE)^21^. Moreover, Wnt1 signaling is indispensable for regulating peripheral ommatidial cell death during the pupal stage in *Drosophila*^22,23^. During eye development, Wnt-mediated apoptosis plays a crucial role in refining the ordered structure of the compound eye by eliminating excess ommatidial cells.

In this study, we report a novel function of Wg/Wnt1 in apoptosis caused by ER stress. We established a *Drosophila* model to study the molecular processes associated with ER stress-triggered cell death and performed an in vivo RNAi screen using this model. RNAi screening, followed by subsequent transcriptome sequencing studies, led to the identification of novel signaling mediators of ER stress-induced apoptosis. These findings were validated using classical gain-in-function and knockdown studies.

## Materials and Methods

### Fly stocks

All *Drosophila melanogaster* stocks were maintained with standard cornmeal medium containing 1.6% yeast, 0.9% soy flour, 6.7% cornmeal, 1% agar, and 7% light corn syrup at 25 °C. The expression of genes in *Drosophila* eyes is mediated through the Gal4/UAS system^24^. The following fly lines, which have been previously described, were used in this study: *GMR*-*GAL4*^25^, *UAS*-*DICER2*^26^, *xbp1*_*p*_ > dsRed^27^, *ninaE*^*G69D*^^28^, and *Rh1-GFP*^29^. *UAS*-*DIAP1* flies were obtained from FlyORF (http://flyorf.ch). The RNAi lines for the in vivo RNAi screen were obtained from the Vienna Drosophila Resource Center, Vienna, Austria (http://stockcenter.vdrc.at). *Erasp* mutant alleles were generated via the CRISPR–Cas9 system. Two gRNA sequences were used to target Cas9 to the *Erasp* coding region. The targeting gRNA sequences used against the coding region of *Erasp* were gRNA1: 5'-AAAACGATCGACTGTCAGCTCGG-3'; gRNA2: 5'-CATTTTGCGAGACGGTATCTTGG-3'.

### Plasmid construction

The coding sequence of Rh-1^P37H^ was amplified from *Rh1* > *ninaE*^*P37H*^ using RT‒PCR^30^ and subcloned into pUAST and pGMR vectors^25^. The GMR-Rh-1^P37H^ line was used for the in vivo RNAi screen. HA-tagged *Erasp*, V5-tagged *Erasp*, HA-tagged *DIAP1, DIAP1*-*EGFP*, and *Dronc-EGFP* were subcloned into a pUAST vector. The Erasp mutant (S228A) was generated using a QuickChange site-directed mutagenesis kit (Stratagene, San Diego, CA, USA). The sequence of the mutant was verified by DNA sequencing.

### Immunoblotting and immunoprecipitation

For immunoblotting, total proteins were extracted from *Drosophila* S2 cells with lysis buffer containing 10 mM Tris-HCl pH 7.5; 1 mM EDTA, pH 8.0; 150 mM NaCl; 1% SDS; and a protease inhibitor cocktail (Roche Diagnostics GmbH., Mannheim, Germany). For immunoblotting, eye imaginal discs were harvested from flies characterized by one of each genotype, and proteins were extracted with 2X Laemmli buffer. After centrifugation at 15,700 × *g* for 10 min at 4 °C, the proteins in the supernatants were resolved by SDS‒PAGE and transferred to polyvinylidene difluoride membranes (Merck Millipore, MA, USA). For the coimmunoprecipitation assay, cells were lysed in lysis buffer (containing 50 mM Tris-HCl, pH 8.0; 150 mM NaCl; 1% NP40; 1 mM DTT; and protease inhibitor; Roche) for 20 min and centrifuged at 15,700 × *g* for 10 min at 4 °C. The supernatants were used for subsequent immunoprecipitation. The immunoprecipitation of DIAP1-EGFP was performed with an anti-EGFP antibody and protein A agarose beads (Bio-Rad Laboratories, Inc., CA, USA). After the beads were washed three times with the same buffer, the immunoprecipitated proteins were analyzed via SDS‒PAGE/immunoblotting. The following antibodies were used in this study:

**Table:** 

Antibody against	Source	1st Antibody dilution	Secondary antibody	2nd Antibody dilution
4C5	DSHB	1:500	Anti-Rabbit Alexa-488 Thermo Fisher Scientific	1:500
ATF4	^31^	1:500	Anti-guinea pig Alexa-488 Thermo Fisher Scientific	1:500
dsRed	Clontech 632496	1:500	Anti-Rabbit Alexa-594 Thermo Fisher Scientific	1:500
V5	Invitrogen R960-25	1:6000	Anti-mouse IgG-HRP Jackson ImmunoResearch	1:5000
HA	Roche 3F10	1:10,000	Anti-rat IgG-HRP Jackson ImmunoResearch	1:5000
EGFP	Invitrogen 6455	1:5000	Anti-Rabbit IgG-HRP Jackson ImmunoResearch	1:5000
α-tubulin	MBL PM054	1:10,000	Anti-Rabbit IgG-HRP Jackson ImmunoResearch	1:5000

### Cell culture and transient transfection

*Drosophila* S2 cells were cultured in Schneider’s *Drosophila* medium (Cat# 21720; Invitrogen, Thermo Fisher Scientific Corp., MA, USA) supplemented with 10% fetal bovine serum (Cat# 16000-044, Invitrogen) and 0.5% penicillin/streptomycin (Cat# 15140-122, Invitrogen). The indicated genes were cloned into a pUAST vector and transfected into cells using Effectene™ (QIAGEN GmbH Company, Hilden, Germany). *Drosophila* S2R+ cells were transfected with a pMK-GFP-Wg plasmid to induce Wg expression and subsequently stimulated by 500 μM CuSO_4_ treatment for the indicated times.

### Immunohistochemistry

All fluorescence images were obtained with a Zeiss LSM710 confocal microscope using a 20X objective lens. The following antibodies were used: rabbit anti-cleaved caspase-3 (diluted 1:50; #9661, Cell Signaling, MA, USA), monoclonal anti-rhodopsin1 (diluted 1:500; Developmental Studies Hybridoma Bank, University of Iowa, IA, USA), rabbit anti-dsRed (diluted 1:500; Clontech Laboratories, Inc., CA, USA), and guinea pig anti-ATF4 (diluted 1:500^32^). In addition, Apoptosis was analyzed by TUNEL staining using an ApopTag Red In Situ Apoptosis Detection kit (S7165, Merck Millipore, MA, USA).

### Analysis of retinal degeneration

As eye pigment can affect retinal degeneration, flies expressing RNA interference (RNAi) against the white-encoding gene (Bloomington stock center #33613, Bloomington, Indiana, IN, USA) were used to analyze retinal degeneration. The flies were reared in vials (20–30 flies in each vial) under permanent lighting conditions at 25 °C. The vials were changed every 2 days. The pseudopupils were quantified with samples placed on a pad under blue fluorescent light after anesthetizing the flies with CO_2_. Cross-sectioning was carried out as previously described^33^, and toluidine blue dye was used to increase the contrast.

### Caspase activity assays

Caspase activity was determined as previously described^34^. Cycloheximide and actinomycin D were purchased from Sigma‒Aldrich, MO, USA (Cat# C7698 and A9415, respectively). Following the indicated treatment, *Drosophila* S2 cells were extracted in caspase assay buffer (50 mM HEPES, pH 7.5; 100 mM NaCl; 1 mM EDTA; 0.1% CHAPS; 10% sucrose; 0.5% Triton X-100; 4% glycerol; 5 mM DTT, and a protease inhibitor cocktail from Roche). Protein concentrations were determined using the Bradford assay. Homogenates (40 μg per assay) were incubated with 100 μM Ac-DEVD-AFC (Cat# ALX-260-032; Enzo Life Sciences, NY, USA) in a final volume of 100 μL of caspase buffer. Fluorescence was measured at excitation and emission wavelengths of 380 nm and 460 nm, respectively, and the reaction was monitored at 30 min intervals for 2 h at 37 °C with a Victor X4 2030 Multilabel Reader (PerkinElmer Inc., MA, USA).

### RNA sequencing

Total RNA was isolated from flies using TRIzol reagent (Invitrogen). A cDNA library was prepared with 1 μg of total RNA from each sample using an Illumina TruSeq mRNA Sample Prep kit (Illumina Inc., CA, USA). The libraries were quantified via qPCRs according to the qPCR Quantification Protocol Guide (KAPA Library Quantification kits for Illumina Sequencing platforms) and qualified using a 2100 Bioanalyzer (Agilent Technologies, Waldbronn, Germany). Subsequently, indexed libraries were sequenced using the HiSeq4000 platform by Macrogen Inc., Seoul, Republic of Korea.

### Read mapping and differential gene expression analysis

Reads were filtered with NGS QC Toolkit^35^ (v.2.3.3) and mapped to the *D. melanogaster* reference transcriptome assembly (dm6) with Bowtie^36^ and RSEM^37^. Differential gene expression analysis was done with expected count data from RSEM results using DEseq2^38^. All the *P-*values were adjusted using multiple testing with a Benjamini‒Hochberg correction and a false discovery rate (FDR) of 5%. Differentially expressed genes were identified via the following thresholds: a fold change >2, a *P*-value < 0.05, and an adjusted *P-*value < 0.05.

### Enrichment analysis

Identification of GO biological process terms and KEGG pathways related to genes that were significantly altered in the RNA-sequencing studies was performed using the Database for Annotation, Visualization, and Integrated Discovery (DAVID)^39^. GO biological process terms exhibiting significant enrichment were selected based on *P*-values < 0.05 and FDR < 0.05.

### RT‒PCR and RT-quantitative PCR

Total RNA was isolated using TRIzol reagent (Invitrogen), and 100 ng of total RNA was transcribed with a ReverTra Ace qPCR RT Kit (Toyobo Co., Osaka, Japan). Quantitative PCR amplification was performed for 40 cycles using TOPreal™ qPCR 2X PreMIX (SYBR Green with high ROX) and a LightCycler® 480 Real-Time PCR System (Roche Diagnostics). *Rp49* was the reference gene used for normalization. Relative quantification of mRNA was performed using the comparative C_Τ_ method. PCR was performed using the following program with iProof™ High-Fidelity DNA polymerase (Cat#1725301; Bio-Rad) on a C1000 Touch™ Thermal Cycler (Bio-Rad): 98 °C for 30 s, 98 °C for 10 s, 57 °C for 30 s, 72 °C for 30 s (for a total of 28 cycles), and 72 °C for 10 min. The primer sequences are listed below:

**Table:** 

Gene name	Forward sequences	Reverse sequences
*CG30090*	GTACAGATGTGGGGCGCTAT	CTCTCCATTGCCCAGAGAAC
*Z600*	TCGACAAATGAAACCAACCA	AACTCATGCTGCTCCTTTGG
*Lim1*	AATATCGGTCGCAGTGAACC	CAGGAACTTGTCCAGGATGG
*pirk*	CGATTCGTATGACGATGACG	GCGTGGAACTTTTCTTGCTC
*CG34034*	AACGGGCACATTCAAACAAT	ATTTTGCTGCCGAATCAATC
*Unc-115b*	GGGCAAGACCTATCACCAGA	CCGGTGTTGGTCACCTTACT
*CR40190*	CTGGGCGAAACTATTTCCAA	CCTCGACAGGAATCCGTTTA
*GstE9*	AGCGTTCATCGGTAATCAGG	CAATCCCACTAGGCTGGAGA
*GstE8*	ACTGCGTGGATCAAGAGGAT	TTGAGGAGGGTCACCAAATC
*CG11854*	AATGGCATTGCTGACATACG	CTCGGCGTAGGTTTGATGAT
*CG31676*	GTGATTTCGCGTTCCTGTTT	GCGGCAGTAGACGGTTTTTA
*rp49*	AGATCGTGAAGAAGCGCACCAAG	CACCAGGAACTTCTTGAATCCGG
*CG16974*	CTTAACTGCGAGCATGTGGA	CGTGGAAGCTCAGTCAACAA
*diap1*	TTGTGCAAGATCTGCTACGG	CCGCCCACATTTTCTTTTTA
*wg*	CAACTTGGCCATTAGCGAGT	ATTTTTGCCCCTCGAGAAGT

### GST pulldown assay

The GST fusion protein, which was expressed in *Escherichia coli* strain BL21, was prepared in GST-binding buffer (50 mM Tris-HCl, pH 8.0; 150 mM NaCl; 0.1 mM EDTA and a protease inhibitor cocktail (Roche Diagnostics GmbH., Mannheim, Germany)) and purified with GSH-Sepharose beads (Amersham, UK). HA-tagged Erasp was expressed in S2 cells, extracted with lysis buffer containing 50 mM Tris-HCl, pH 8.0; 150 mM NaCl; 1% Triton X-100;0.1% SDS; and 0.5% sodium deoxycholate and incubated with purified GST fusion protein-bound beads for 4 h at 4 °C. The beads were washed extensively with the same lysis buffer to remove nonspecific proteins. The extracted proteins were boiled in 2X Laemmli buffer and subjected to SDS‒PAGE/immunoblotting.

### Scanning electron microscopy

Standard procedures were used for sample preparation, including fixation in 2% glutaraldehyde, dehydration, and drying in CPD (HCP-2, HITACHI, Japan). Platinum was used to coat adult flies, and images were taken at 180 x and 500 x magnification.

## Results

### Wg/Wnt1 knockdown suppresses the Rh-1^P37H^ overexpression-induced phenotype in *Drosophila*

**Fig. 1:**
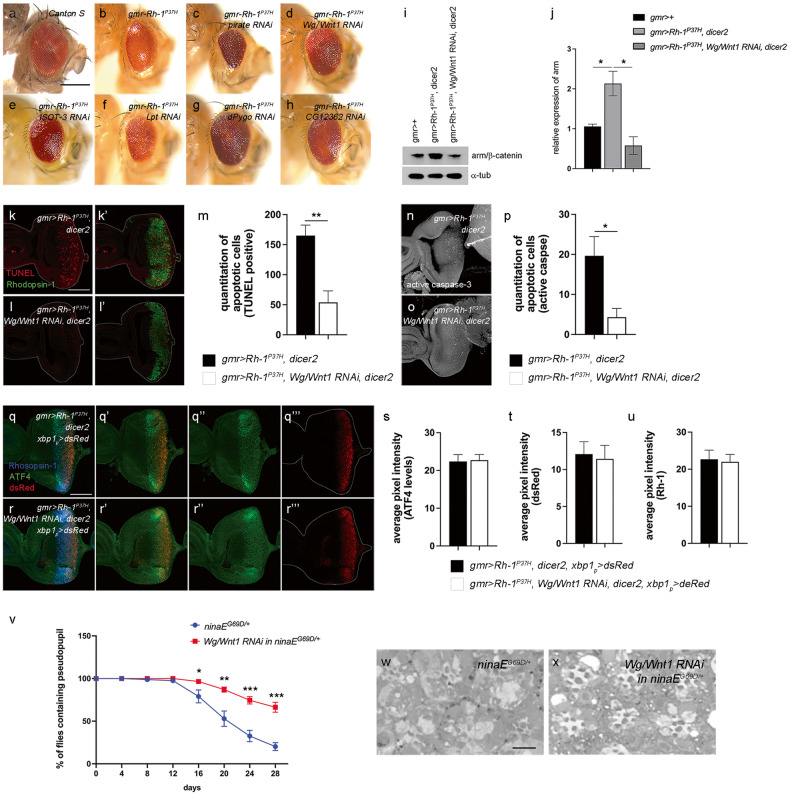
Wg/Wnt1 is vital for Rh-1^P37H^-induced apoptosis of *Drosophila* retinal cells. **a**–**h** External adult eyes misexpressing Rh-1^P37H,^ together with genetic modifiers were identified through an in vivo RNAi screen. **a** Canton S. **b** Rh-1^P37H^-expressing flies without an inverted repeat transgene have abnormally small eyes with shinny surfaces. **c**–**h** Genetic modifiers. **c** *Pirate*, **d** *Wg/Wnt1*, **e** *ISOT-3*, **f** *Lpt*, **g** *dPygo*, and **h** *CG12362*. **i**, **j** The levels of arm/β-catenin in eye discs. The accumulation of arm/β-catenin due to Rh-1^P37H^ misexpression was reduced in Wg/Wnt1 knocked down eye discs (**i**). Comparison of the relative expression of the arm in these eye imaginal discs (*n* = 5) (**j**). **k**, **l** Apoptosis of Rh-1^P37H^-misexpressing eye discs as assessed using TUNEL staining (red). Rh-1^P37H^-triggered apoptosis (**k**) was suppressed by the knockdown of *Wg/Wnt1* (**l**). Green fluorescence indicates anti-Rh-1 staining. **m** Comparison of the numbers of apoptotic cells between those presented in (**k**) and (**l**) (*n* = 3). **n**, **o** Caspase activation due to misexpression of Rh-1^P37H^ was suppressed under *Wg/Wnt1* knockdown conditions. Larval eye imaginal discs were labeled with an anti-cleaved caspase antibody (white) to assess cell death. (**p**) Comparison of the numbers of active caspase-positive cells in (**n**) and (**o**) (*n* = 3). **q**, **r** The degree of ER stress was analyzed with the ER stress reporters xbp1_p_ > dsRed and anti-ATF4. The transcriptional activation of XBP1 on misexpression of Rh-1^P37H^ was assessed by dsRed expression (red). The degree of xbp1p > dsRed activation and ATF4 induction by Rh-1^P37H^ misexpression was similar between (**q**) and (**r**). Green: ATF4, blue: rhodopsin-1. **s** Quantification of ATF4 induction by Rh-1^P37H^ misexpression based on (Q” and R”) (*n* = 9). **t** The induction of dsRed was measured with the NIH ImageJ program*. Wg/Wnt1* knockdown did not affect the degree of XBP1 pathway activation (*n* = 9). **u** The levels of Rh-1 were similar between (**q**) and (**r**) (*n* = 5). **v** Quantification of photoreceptor cell degeneration using a pseudopupil assay. For each genotype, the percentage indicates the number of flies with an intact Rh-1 > GFP pattern. Knockdown of *Wg/Wnt1* suppressed the course of retinal degeneration in *ninaE*^*G69D/+*^ flies (*n* = 9). **w**, **x** Representative images of 32-day-old adult eye tangential sections with downregulated Wg/Wnt1 expression in the *ninaE*^*G69D/+*^ background. Error bars indicate ±S.E.M. *P-*values were determined using Student’s *t*-tests. *P < 0.05, **P < 0.01, and ***P < 0.001. The scale bar in (**a**) corresponds to 50 μm in (**a**–**h**) and 100 μm in (**k**) and (**q**). The scale bar in (**w**) corresponds to 5 μm (**w**, **x**).

In previous studies, we developed an in vivo model of ER stress-induced apoptosis in *Drosophila* that harbors a mutant rhodopsin-1 (Rh-1) allele, Rh-1^G69D^, and we identified several mediators of ER stress-induced apoptosis^31,40^. In this study, we modified the model to express the mutant Rh-1 allele Rh-1^P37H^, which is the equivalent of mammalian Rh-1^P23H^ and the most common autosomal dominant retinitis pigmentosa (ADRP)-related mutation in humans^41,42^. Overexpression of the Rh-1^P37H^ mutant in larval eye imaginal discs caused ER stress, as validated by three independent reporters (Supplementary Fig. 1), namely, xbp1p>dsRed^27^, ATF4^31^, and XBP1-EGFP^43^. Moreover, Rh-1^P37H^-overexpressing flies presented with an abnormal external eye phenotype (Fig. 1a, b). This phenotype was suppressed by CDK5 knockdown (Supplementary Fig. 2), which was previously described as a mediator of ER stress-induced apoptosis^31^. All the detectable ER stress-related phenotypes were quite similar to those previously reported in Rh-1^G69D^ mutant allele-harboring flies^31^.

Based on the external eye phenotype, a subsequent in vivo RNAi screen was conducted to identify genes that are critical for Rh-1^P37H^ allele-associated toxicity. Specifically, we screened 354 inverted repeat transgenes that target ubiquitin-related genes (NYU-DRSC UBIQ, https://fgr.hms.harvard.edu/drsc-focused-sub-libraries) (Supplementary Table 1). Knockdown of the genes resulted in aggravation of the eye phenotype in most flies (Supplementary Fig. 3). In particular, our results demonstrated that six *Drosophila* lines showed profound suppression of adult eye phenotype (Fig. 1c–h), with two fly lines (VDRC104579 and VDRC100724) sharing the Wg/Wnt1 signaling pathway in common: one line (VDRC104579) targeted *Drosophila* Wg/Wnt1 and the other line (VDRC100724) targeted Pygopus, which is a key nuclear component of the Wnt signaling pathway. The other suppressors of Rh-1 toxicity were found to be Pirate, ISOT-3, Lpt, and CG12362 (Fig. 1c, e, f, and h).

Wg/Wnt1 is the main regulatory pathway in cell proliferation and differentiation during development and adult tissue homeostasis. In addition, aberrant Wnt signaling has often been attributed to multiple human diseases, including cancers^19,44,45^. Investigation of the knockdown efficiency of RNAi targeting Wg/Wnt1 as measured through quantitative RT‒PCR revealed that Wg/Wnt1 expression was knocked down by ~90% (Supplementary Fig. 4a). To verify the involvement of the Wg/Wnt1 signaling pathway in Rh-1^P37H^ overexpression-induced ER stress, we examined the accumulation of armadillo/β-catenin, which is stabilized by Wnt activation^20^. The accumulation of armadillo/β-catenin on misexpression of Rh-1^P37H^ was decreased when Wg/Wnt1 was knocked down (Fig. 1i, j), indicating that the Wg/Wnt1 signaling pathway is activated by ER stress. Subsequent loss-of-function studies revealed that Wg/Wnt1 knockdown itself did not affect the external eye phenotype (Supplementary Fig. 4b, c). In addition, Wg/Wnt1 knockdown did not influence the cell death phenotype induced by p53 overexpression (Supplementary Fig. 4d, e), indicating that Wg/Wnt1 mediates specific apoptotic signaling in response to mutant Rh-1 expression. However, examination of cell death in Rh-1^P37H^-expressing eye imaginal discs revealed that the proportion of TUNEL-positive cells was significantly reduced, by 70%, compared with that in control discs, indicating that *Wg/Wnt1* is essential for apoptosis in Rh-1^P37H^-overexpressing eye imaginal discs (Fig. 1k–m). Furthermore, an investigation of cellular caspase activity revealed that active caspase staining was consistently reduced in Wg/Wnt1*-*knockdown eye discs (Fig. 1n–p).

To determine whether the suppression of apoptosis by *Wg/Wnt1* knockdown is attributed to the reduction in overall ER stress, we used the ER stress reporter xbp1_p_ > dsRed^27^ in eye imaginal discs misexpressing Rh-1^P37H^ and labeled them with an anti-ATF4 antibody. Our results showed that the overall intensities of the xbp1_p_ > dsRed reporter and the levels of induced ATF4, and Rh-1^P37H^ expression upon misexpression Rh-1^P37H^ were similar between the control and the *Wg/Wnt1*-knockdown groups (Fig. 1q–u). These results indicate that Wg/Wnt1 specifically mediates proapoptotic signaling without affecting the overall levels of ER stress. To test whether the Wg/Wnt1 pathway is relevant to age-dependent disease progression, we examined retinal degeneration in a *Drosophila* model of ADRP, in which a mutant allele of the Rh-1 gene, *ninaE*^*G69D*^, causes age-dependent retinal degeneration^28,46^. An Rh-1 > GFP reporter-based pseudopupil assay was performed to monitor retinal degeneration in live flies. The results demonstrated that the *ninaE*^*G69D/+*^ flies started to lose their pseudopupil at 12 d in a progressive manner, with only 20% of these flies presenting with an intact pseudopupil at 28 d after eclosion (Fig. 1v, blue line). However, the knockdown of *Wg/Wnt1* in photoreceptor cells using the Rh-1 driver significantly delayed the time course of retinal degeneration, with 66% of the examined flies exhibiting intact Rh-1 > GFP patterns at 28 d (Fig. 1v, red line). The ommatidia in the retinas of 32-day-old *ninaE*^*G69D/+*^ flies were mostly disorganized (Fig. 1w), which was rescued by *Wg/Wnt1* knockdown (Fig. 1x). Consistently, the knockdown of *dPygo* identified with Wg/Wnt1 from in vivo RNAi screen as a modulator of Rh-1^P37H^ toxicity (Fig. 1g) suppressed apoptosis triggered by Rh-1^P37H^ overexpression (Supplementary Fig. 5a–d).

To investigate whether the IRE1 pathway contributes to Wg/Wnt1-mediated apoptosis, we examined the degree of cell death by analyzing the adult eye phenotype. Knocking down *ire1* using RNAi aggravated the eye phenotype caused by Rh-1^P37H^ misexpression. However, the eye phenotype was largely restored when *ire1* and *Wg/Wnt1* were knocked down together (Supplementary Fig. 6). These results indicate that the IRE1 pathway protects against misfolded protein accumulation in the ER rather than simply activating proapoptotic signaling proteins. Altogether, these results indicate that the Wg/Wnt1 signaling pathway mediates the apoptosis of retinal cells under ER stress conditions and plays an essential role in the progression of retinal degeneration in the presence of ER stress.

### RNA sequencing reveals the association of multiple transcription targets of Wg/Wnt1 in ER stress-mediated apoptosis of retinal cells in the *Drosophila* ADRP model

**Fig. 2:**
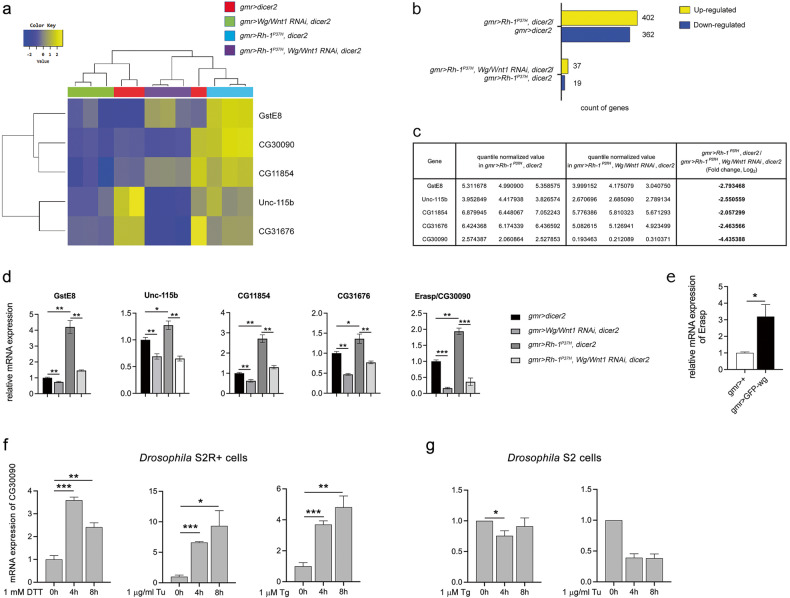
RNA sequencing reveals the association of multiple transcription targets of Wg/Wnt1 in ER stress-mediated apoptosis of ADRP-afflicted *Drosophila* retinal cells. **a** Heatmap of the two-way hierarchical clustering showing five differentially expressed genes (fold change > 2, *P* < 0.05) in *GMR* > *DICER2* (red, *n* = 3), *GMR* > Wg/*Wnt1 RNAi, DICER2* (green, *n* = 3), *GMR* > *Rh-1*^*P37H*^*, DICER2* (blue, *n* = 3), and *GMR* > *Rh-1*^*P37H*^*, Wg/Wnt1 RNAi, DICER2* flies (purple, *n* = 3). **b** The number of upregulated (yellow) or downregulated (blue) genes in flies with the indicated genotypes (fold change > 2, *P* < 0.05). **c** Quantile-normalized values of select genes that were regulated by Wg/Wnt1 under ER stress in the indicated genotypes according to RNA-seq analysis (refer to Supplemental Table 4). **d** Quantitative RT‒PCR analysis of selected genes in eye imaginal discs with the indicated genotypes; the levels were normalized to those of *rp49* and presented relative to those of *GMR* > *DICER2*. **e** Normalized *Erasp* mRNA expression after GFP-wg overexpression in eye imaginal discs. **f** ER stress induced in response to independent treatment with chemicals, such as DTT (1 mM), Tu (1 µg/mL), and Tg (1 µM), triggered the induction of *Erasp* at the transcript level in *Drosophila* S2R+ cells, whereas this induction of *Erasp* by Tg (1 μM) or Tu (1 µg/mL) was absent in *Drosophila* S2 cells (**g**). *P*-values were determined using Student’s *t*-tests. *P < 0.05, **P < 0.01, and ***P < 0.001.

To identify the signaling pathway mediated by Wg/Wnt1 under ER stress, we analyzed the differentially expressed genes (DEGs) that were identified from the RNA-sequencing data of eye imaginal discs with the following genotypes: *GMR* > *DICER2* (red), *GMR* > *Wg/Wnt1 RNAi, DICER2* (green), *GMR* > *Rh-1*^*P37H*^*, DICER2* (blue), and *GMR* > *Rh-1*^*P37H*^*, Wg/Wnt1 RNAi, DICER2* (purple) (Fig. 2a). The analysis identified 1134 transcripts that were differentially expressed in all samples (Supplementary Table 2, *P* < 0.05), and only the transcripts with a fold change >2 were selected for the subsequent analysis. When we compared the gene expression pattern between control discs (*GMR* > *DICER2*) and Rh-1^P37H^-misexpressing imaginal discs (*GMR* > *Rh-1*^*P37H*^*, DICER2*), a total of 764 genes were shown to be differentially expressed. Of these DEGs, 402 and 362 genes were up- and downregulated upon Rh-1^P37H^ misexpression, respectively (Fig. 2b and Supplementary Table 3). A subsequent Gene Ontology (GO) analysis categorized these DEGs into enriched functional groups, which included genes encoding proteins for axon guidance, development, and protein ubiquitination (Supplementary Fig. 7).

To identify the transcriptional targets of Wg/Wnt1 during ER stress, we compared the gene expression between two samples, *GMR* > *Rh-1*^*P37H*^*, Wg/Wnt1 RNAi, DICER2* and *GMR* > *Rh-1*^*P37H*^*, DICER2*. The analysis revealed that *Wg/Wnt1* knockdown resulted in the down- and upregulation of 19 and 37 transcripts, respectively, compared with control fly lines expressing endogenous levels of *Wg/Wnt1* (Fig. 2b and Supplementary Table 4). Consistent with the RNA-sequencing analysis data (Fig. 2a, c), the qPCR data showed that the expression of the genes *GstE8*, *Unc-115b*, *CG11854*, *CG31676*, and *CG30090* was regulated by Wg/Wnt1 signaling (Fig. 2d). In addition, we found that all the genes we investigated were upregulated by ER stress, i.e., upon misexpression of Rh-1^P37H^ compared with their expression in control wild-type flies. Given that *CG30090* is the most highly ranked gene identified in the RNA sequencing studies and that its cellular function has not been fully characterized, we decided to further explore the novel functions of CG30090 in ER stress-mediated cell death in *Drosophila*.

To verify that the gene expression of CG30090 is regulated by Wg/Wnt1 in vivo, we misexpressed *GFP*-*wg* in eye discs and found that the CG30090 mRNA expression level was significantly increased by Wg/Wnt1 induction (Fig. 2e). To evaluate whether the expression of CG30090 is regulated by ER stress, we exposed *Drosophila* S2R+ cells—which express a Wg receptor, *Drosophila frizzled 2* (*Dfz2*)^47^—to ER stress-inducing chemicals, namely, dithiothreitol (DTT), tunicamycin (Tu), and thapsigargin (Tg). As shown in Fig. 2f, the expression of CG30090 at the transcript level was increased in response to ER stress. However, thapsigargin and tunicamycin failed to induce the expression of CG30090 in *Drosophila* S2 cells—which do not express *Dfz2*— (Fig. 2g), indicating that the expression of *CG30090* in response to ER stress is regulated by Wg/Wnt1 signaling. As *CG30090* expression was identified under ER stress in the present study, we termed this gene ER stress-associated serine protease (*Erasp*) hereafter.

### Erasp is required for ER stress-induced apoptosis of retinal cells in the *Drosophila* ADRP model

**Fig. 3:**
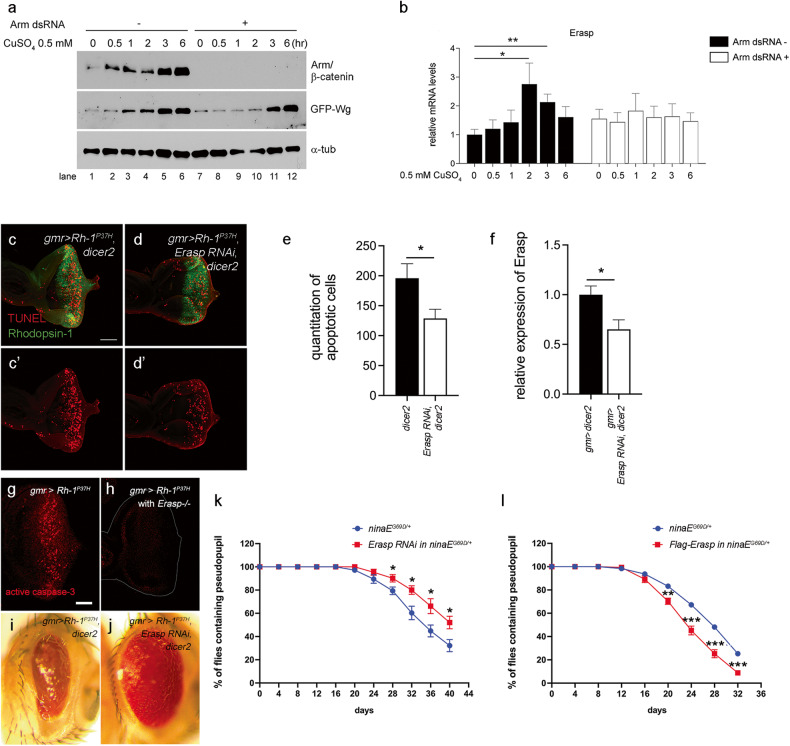
Erasp mediates Rh-1 toxicity in *Drosophila* retinal cells. **a** The induction of Arm protein expression by GFP-Wg. *Drosophila* S2R+ cells transfected with pMK-GFP-Wg were incubated with CuSO_4_ for the indicated times. Lanes 1-6: control S2R+ cells, Lanes 7-12: S2R+ cells pretreated with dsRNA targeting arm. **b** The mRNA expression of *Erasp* after overexpression of Wg was analyzed in cells pretreated without or with dsRNA targeting arm. **c**–**e** Apoptosis in eye discs as assessed through TUNEL staining (red). Substantial apoptosis caused by Rh-1^P37H^ misexpression (**c'**) was significantly suppressed in *Erasp* knockdown in eye discs (**d'**). **e** The number of apoptotic cells in representative images was quantified (*n* = 9). Error bars indicate ± S.E.M. **f** Knockdown efficiency of Erasp RNAi in eye discs. Quantitative RT‒PCR results based on an analysis of dissected third-instar larval eye discs are shown. RNAi was expressed in tissues with the *gmr*-*gal4* driver. The error bars indicate ± S.E.M. **g**, **h** The levels of apoptosis were assessed by labeling active caspase-3 in an *Erasp*-/- background. Misexpression of Rh-1^P37H^ led to substantial apoptosis (**g**), which was suppressed in the *Erasp*-/- mutant (**h**). **i**, **j** External adult eyes. The degree of eye ablation as a result of Rh-1^P37H^ misexpression (**i**) was suppressed in an *Erasp*-knockdown fly (**j**). **k** Quantification of age-related retinal degeneration in *ninaE*^*G69D/+*^ flies. Knockdown of *Erasp* suppressed retinal degeneration in this model (*n* = 7). **l** The overexpression of Erasp enhanced late-onset retinal degeneration of *ninaE*^*G69D/+*^ (*n* = 8). *P*-values were determined using Student’s *t-*tests. *P < 0.05, **P < 0.01, and ***P < 0.001. The scale bar in (**c**) represents 100 μm for (**c**) and (**d**) and that in (**g**) represents 50 μm for (**g**) and (**h**).

Wnt signaling pathways are divided into two types: canonical and noncanonical pathways^20^. The involvement of β-catenin characterizes the canonical pathway, whereas the noncanonical pathway functions independently of β-catenin. In the canonical pathway, upon Wnt activation, β-catenin is stabilized and transported from the cytosol to the nucleus, where it recruits TCF/LEF family cofactors to regulate the expression of target genes that are related to cell proliferation and differentiation. As we identified *Erasp* as a potential transcriptional target of Wg/Wnt1, we investigated whether the induction of *Erasp* is mediated by the canonical or noncanonical Wg/Wnt1 pathway. To this end, we induced the expression of Wg using the copper-inducible metallothionein promoter (pMT) in *Drosophila* S2R+ cells. Subsequent studies revealed that the expression of *Erasp* was increased by the overexpression of *Wg*. However, this increase in expression was abolished when the armadillo/β-catenin expression was knocked down using dsRNA targeting armadillo in *Drosophila* S2R+ cells, indicating that the induction of *Erasp* expression by Wg/Wnt1 activation is mediated through the canonical pathway (Fig. 3a, b). Furthermore, the noncanonical Wg/Wnt1 signaling pathway was also affected by ER stress, as the Rh-1^P37H^ misexpression-induced activation of JNK, which is a mediator of the noncanonical Wnt signaling pathway, was suppressed when the Wg/Wnt1 level was decreased (Supplementary Fig. 8).

We further investigated whether *Erasp* plays a role in cell death caused by Rh-1^P37H^-associated toxicity. Consistent with the results obtained with Wg/Wnt1-knockdown *Drosophila* lines, knocking down *Erasp* in *Drosophila* eye imaginal discs suppressed the apoptosis that had been triggered by Rh-1^P37H^ misexpression (Fig. 3c–e), even when the knockdown efficiency of *Erasp* RNAi was ~35% (Fig. 3f). This effect was verified using CRISPR‒Cas9-engineered *Erasp*-mutant flies (Supplementary Fig. 9). The *Drosophila Erasp* mutants were viable and exhibited no developmental defects. Accordingly, the substantial apoptosis induced by Rh-1^P37H^ misexpression, measured by cleaved caspase-3 antibody labeling, was significantly reduced in flies with an Erasp-/- background (Fig. 3g, h). Moreover, the Rh-1^P37H^ misexpression phenotype was suppressed in an *Erasp*-knockdown fly line (Fig. 3i, j). To investigate whether Erasp affects disease progression in a *Drosophila* model of ADRP, we performed a pseudopupil assay and found that knockdown of *Erasp* in photoreceptors delayed age-related retinal degeneration in the *Drosophila* model of ADRP (Fig. 3k). In contrast, overexpression of Erasp enhanced photoreceptor cell degeneration in the ADRP model (Fig. 3l). These results indicate that Erasp is involved in Rh-1^P37H^-induced apoptosis of retinal cells in the *Drosophila* model of ADRP.

### Erasp mediates caspase-dependent apoptosis

**Fig. 4:**
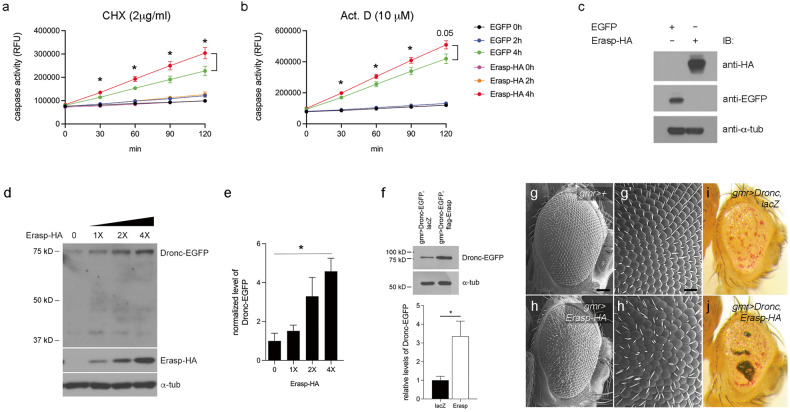
Erasp is a novel mediator of caspase-dependent apoptosis. **a**–**c** In vitro caspase activity was measured in control (*EGFP*-overexpressing S2 cells) or *Erasp-HA*-overexpressing cells. Cell death was triggered by CHX (**a**) or Act. D (**b**). The overexpression of *Erasp* enhanced the caspase activity induced by CHX or Act. D. **c** The control blot for (**a**) and (**b**). The expression of Erasp was measured with an anti-HA antibody. EGFP was used as a control gene. **d** The levels of Dronc were increased in an Erasp-HA dose-dependent manner. The amounts of Dronc fused to EGFP were measured by anti-EGFP antibody. α-Tubulin was used as a loading control. **e** The normalized band intensities of the gels shown in (**d**) were quantified, and the data are shown in the graph. **f** The levels of Dronc in eye imaginal discs. The level of Dronc, as measured with an anti-EGFP antibody, was significantly increased by the overexpression of Erasp in vivo. The graph indicates the relative levels of Dronc-EGFP (*n* = 3). **g**–**j** Representative eye phenotypes of flies expressing the indicated genes. The expression of genes was driven by the *GMR*-*GAL4* driver. Scanning EM image of an external adult eye with Erasp misexpression shows a rough eye phenotype, missing bristles, and abnormal bristle production (**h**, **h'**) compared to those in control (**g**, **g'**). The abnormal eye phenotype of *Dronc*-expressing flies (**i**) was aggravated by the coexpression of *Erasp* (**j**). *P*-values were determined using Student’s *t*-tests. *P < 0.05. The scale bar in (**g**) represents 60 μm for (**g**) and (**h**) and that in (**g'**) represents 20 μm for (**g'**) and (**h'**).

To verify the apoptotic effect of Erasp, we examined caspase activity in *Drosophila* S2 cells overexpressing *Erasp*. Accordingly, we treated these cells with the apoptosis inducers cycloheximide (CHX) and actinomycin D. Further analysis revealed that stimulation of HA-tagged Erasp-overexpressing cells with CHX for 4 h significantly increased their caspase activity compared with that in control cells (EGFP-overexpressing cells) (Fig. 4a, c). Similarly, we performed fluorometric assays to measure caspase activity in Erasp-overexpressing cells, and the results revealed significantly higher cleavage of a synthetic DEVD substrate than that in control cells after treatment with actinomycin D for 4 h (Fig. 4b, c); these results indicated that Erasp mediates apoptosis via the activation of caspases.

In *Drosophila*, apoptotic stimuli induce Apaf-1 and the initiator caspase Dronc to form an apoptosome complex, which ultimately cleaves Dronc^48–50^. As Erasp is a serine protease with a trypsin domain, we examined the cleavage products of Dronc in the presence of Erasp to determine whether Erasp is involved in the proteolytic cleavage of Dronc. Our results showed that the levels of cleaved Dronc fragments were not significantly different, even in the presence of high concentrations of Erasp, compared with those under control conditions without Erasp (Fig. 4d). In contrast, we found that the levels of Dronc, as determined by the detection of the fusion protein—Dronc-EGFP—using an anti-EGFP antibody, gradually increased as the amount of Erasp was increased (Fig. 4d, e), indicating that Erasp was potentially involved in the stabilization of Dronc. To further validate these findings, we examined an in vivo model for the overexpression of *Erasp* using the eye-specific gene expression driver *GMR-GAL4*. Coexpression of Dronc-EGFP with Flag-tagged Erasp significantly increased the stability of Dronc compared to the control without Erasp expression (Fig. 4f). In addition, our analysis revealed that overexpressing *Erasp* resulted in a slightly rough eye phenotype, missing bristles, and abnormal bristle production, suggesting that an increased amount of Erasp alone can trigger apoptosis in vivo (Fig. 4g, g', h and h'). The combined expression of Dronc and HA-tagged Erasp further promoted the apoptosis of retinal cells, resulting in a severe rough eye phenotype, pigment loss, and eye abnormalities compared with those in *Drosophila* lines expressing Dronc alone (Fig. 4i, j). Altogether, these results indicate that Erasp is a novel mediator of the apoptosis pathway.

### Erasp controls apoptosis by degrading DIAP1

**Fig. 5:**
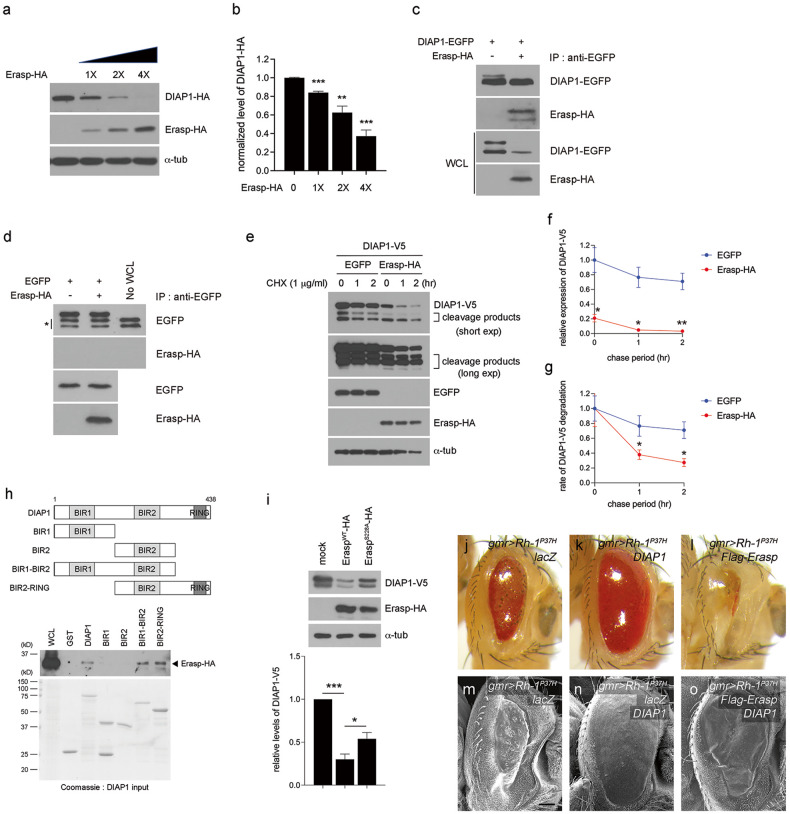
Erasp regulates the degradation of DIAP1. **a** *Drosophila* S2 cells were cotransfected with *Erasp-HA* and *DIAP1-HA*. Whole-cell lysates were immunoblotted to determine DIAP1 and Erasp expression levels. **b** Graph showing quantified and normalized band intensities from the immunoblot (**a**). Error bars show ± S.E.M. **c** Erasp physically interacts with DIAP1 in *Drosophila* S2 cells. DIAP1 was immunoprecipitated with an anti-EGFP antibody, and its interaction partner, Erasp, was detected through its HA epitope. **d** EGFP does not interact with Erasp. An asterisk indicates the light chain of the antibody. WCL: Whole-cell lysate. **e** DIAP1 degradation was accelerated by the overexpression of *Erasp*. HA-tagged *Erasp* and V5-tagged *DIAP1* were transiently cotransfected into S2 cells. Then, chase experiments were performed at the indicated time points after the addition of 1 μg/mL cycloheximide (CHX) at time zero. α-Tub was used as a loading control. **f** Quantification of DIAP1-V5 signals in (**e**) normalized to those of endogenous α-tubulin. **g** Graphs show the rate of DIAP1 degradation. The DIAP1 level at 0 h chase in each panel of (**e**) was set to 1. **h** GST pulldown assay. GST-DIAP1 and truncation mutants purified using GST-Sepharose beads were incubated with HA-tagged Erasp-overexpressing S2R+ cells for 4 h at 4 °C. The bead complexes were separated by SDS‒PAGE and immunoblotted using an anti-HA antibody. **i** Compared to the effect of wild-type Erasp (Erasp^WT^-HA) on DIAP1 stability, catalytically inactive Erasp (Erasp^S228A^-HA) restored the levels of DIAP1. The graphs show the normalized band intensities from the blot. **j**–**l** External adult eye phenotypes caused by Rh-1^P37H^ overexpression, together with the indicated genes. **m**–**o** Scanning EM image of external adult eyes. The expression of Rh-1^P37H^ in larval eye discs resulted in small adult eyes with abnormally smooth surfaces (**m**), and this effect was partially reversed by DIAP1 coexpression (**n**). The eye phenotype rescued by DIAP1 expression was aggravated by Erasp coexpression (**o**). The scale bar in (**m**) represents 60 μm for (**m**–**o**). Error bars indicate ± S.E.M. *P*-values were determined using Student’s *t*-tests. *P < 0.05, **P < 0.01, and *** *P* < 0.001.

*Drosophila* inhibitor of apoptosis protein 1 (DIAP1) is a key component of the apoptosis in *Drosophila* that suppresses the activation of caspases^51–53^. DIAP1 uses the E3 ubiquitin ligase activity of its RING domain to ubiquitylate Dronc directly, and it is autoubiquitylated in response to proapoptotic stimuli^54–56^. It has been reported that *Drosophila* OMI/HTRA2, which is a serine protease that has been characterized as an IAP antagonist, degrades DIAP1^57,58^, thereby triggering apoptosis. Since Dronc was found to be stabilized by Erasp, we investigated whether Erasp is also involved in the degradation of DIAP1, similar to the effect of OMI/HTRA2. Accordingly, HA-tagged DIAP1 was cotransfected with Erasp-HA into *Drosophila* S2 cells, and subsequent studies revealed that DIAP1 expression was downregulated when coexpressed with *Erasp* in a dose-dependent manner (Fig. 5a, b). Next, we examined the interaction between Erasp and DIAP1 using coimmunoprecipitation assay. Our results demonstrated an evident interaction between DIAP1 and Erasp when *DIAP1* fused with *EGFP* was coexpressed with HA-tagged *Erasp* in *Drosophila* S2 cells (Fig. 5c); notably, Erasp alone did not interact with EGFP (Fig. 5d).

To further determine that Erasp degrades DIAP1, we performed a chase experiment using cycloheximide (CHX), which inhibits protein synthesis by interfering with the translocation step during translation. Consequently, we observed that the levels of V5-tagged DIAP1 decreased radically when Erasp-HA was overexpressed in *Drosophila* S2 cells (Fig. 5e, f). Despite the limited detection of very small DIAP1 fragments, the cleavage products and amounts of small DIAP1 fragments were higher in *Erasp*-overexpressing cells than in control cells expressing endogenous levels of *Erasp* (Fig. 5e). Furthermore, the chase experiment confirmed that the overexpression of *Erasp* facilitated the degradation of DIAP1, as suggested by the rapid decrease in the levels of DIAP1 compared with that in the control cells expressing endogenous levels of *Erasp* (Fig. 5g). As the mRNA levels of *DIAP1* were comparable in the mock- and *Erasp*-HA-overexpressing S2 cell lines (Supplementary Fig. 10), we concluded that Erasp actively regulates the expression level of DIAP1 at the posttranslational level.

To determine the domain involved in DIAP1 binding to Erasp, we performed a GST pulldown assay with various DIAP1 truncation mutants fused with GST in Erasp-overexpressing S2 cells. We found that all three domains were required for its interaction with Erasp and that at least two of these domains strengthened the interaction between the two proteins (Fig. 5h). Furthermore, when the catalytically active serine-228 residue in Erasp was mutated, the amount of DIAP1 was significantly restored (Fig. 5i), indicating that Erasp regulates the stability of DIAP1 via its serine protease activity. Finally, the effect of Erasp on DIAP1 degradation was validated using in vivo eye tissues. The results showed that the Rh-1^P37H^ misexpression-induced phenotype (Fig. 5j, m) was evidently suppressed by *DIAP1* coexpression (Fig. 5k). In contrast, the eye abnormalities caused by the misexpression of Rh-1^P37H^ were further exacerbated upon *Erasp* coexpression (Fig. 5l). These findings indicate that the activity of Erasp is elevated under stress conditions, such as ER stress that is induced by misexpression of Rh-1^P37H^. Moreover, the Rh-1^P37H^ misexpression-driven eye phenotype was suppressed by *DIAP1* overexpression (Fig. 5n), and this effect was reversed and aggravated with the coexpression of *Erasp* (Fig. 5o), confirming that Erasp inhibits the antiapoptotic function of DIAP1. Altogether, these results indicate that Erasp mediates caspase-dependent ER stress-induced apoptosis in *Drosophila* retinal cells by degrading DIAP1.

## Discussion

**Fig. 6:**
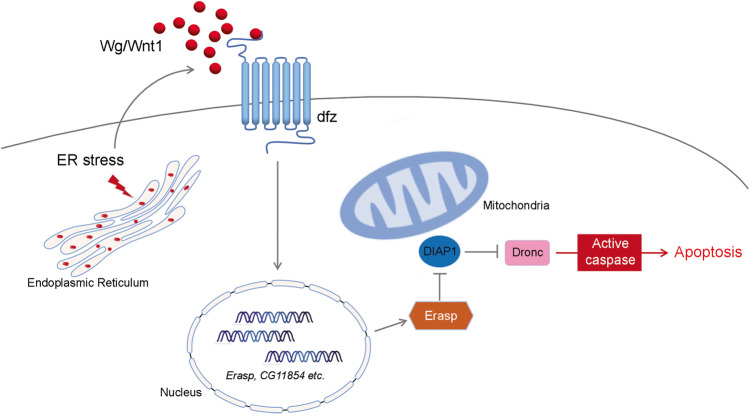
A model of ER stress-induced apoptosis. The accumulation of misfolded proteins in the ER activates Wg/Wnt signaling, which induces the expression of genes, including *Erasp*, through the canonical pathway. Erasp then triggers the degradation of DIAP1 and stabilizes Dronc, ultimately enhancing apoptosis.

In the present study, we have reported that Wg/Wnt1 and Erasp were previously unrecognized regulators of ER stress-induced apoptosis in *Drosophila*. Although several studies have described the Wnt signaling pathway as an essential pathway that regulates growth and is associated with development and cancer, to the best of our knowledge, none of those studies have identified Wnt as a mediator of ER stress-induced cell death. Our data show that the knockdown of Wg/Wnt1 and *Erasp*, a transcriptional target of Wg/Wnt1, suppressed proapoptotic signaling in a *Drosophila* model of ADRP. Furthermore, we have demonstrated that Erasp stabilizes Dronc by triggering DIAP1 degradation, thus promoting cell death; therefore, Erasp acts as a mediator of the novel apoptosis signaling pathway that is associated with ER stress-induced cell death (Fig. 6).

The Wnt signaling pathway is highly conserved across various species, from *Drosophila* and *Xenopus* to humans, and it controls multiple biological processes during development and adult tissue/organ homeostasis^19,44^. Wnt proteins, which are secretory glycoproteins, are targeted to the ER and are lipid-modified by Porcupine, an acyl transferase. This lipid modification of Wnt proteins is essential for their secretion as well as their efficient signaling. In the Golgi apparatus, the transmembrane protein Wntless/Evi (Wls) binds to the modified Wnt proteins, facilitating their translocation to the plasma membrane. However, the silencing of Porcupine or Wls leads to defects in Wg/Wnt1 secretion, causing the accumulation of Wg/Wnt1 in the ER and resulting in ER stress^59^. Accordingly, considering the cellular location of their synthesis and the structural properties of Wnt proteins, it is not surprising that Wnt proteins are involved in ER stress. Our data indicate that ER stress induced by the misexpression of Rh-1^P37H^ activates the Wg/Wnt1 signaling pathway to promote the apoptosis of *Drosophila* retinal cells. However, the upstream molecular signals that activate the Wg/Wnt1 pathway were not clarified through our experimental system. A recent study has indicated that the expression of Porcupine and Wls increases under various stresses, including ER stress^60^. Thus, we hypothesized that the increased levels of Porcupine and Wls might promote the secretion of Wnt and thus induce pro-apoptotic gene expression under excess ER stress.

In this study, we propose the function of Erasp, which is classified as a serine protease, as a novel IAP antagonist in *Drosophila*. The expression of *Erasp* induces cell death, as evidenced by the appearance of a weak rough eye phenotype upon *Erasp* misexpression (Fig. 4h, h’). In addition, elevated *Erasp* expression enhanced caspase activity without apoptotic stimuli, further supporting our hypothesis (Supplementary Fig. 11). Thus, the expression or activity of Erasp should be tightly regulated to allow cell survival. We also hypothesized that Erasp might perform functions similar to those of *Drosophila* OMI/HTRA2, which is a known mitochondria-localized IAP antagonist^57,58^. The removal of the mitochondrial targeting sequence in *Drosophila* OMI/HTRA2 exposes an IAP-binding motif (IBM) that is vital for its interaction with IAP, and the processed *Drosophila* Omi/HtrA2 then interacts with DIAP1. As the Erasp protein sequence does not contain a mitochondrial targeting sequence (MTS), such as that in *Drosophila* OMI/HTRA2, it is logical to conclude that Erasp proteins are not likely to be transported to mitochondria. Thus, the activity of Erasp is controlled in quite a different manner from the regulatory mechanism of *Drosophila* OMI/HTRA2. Considering that the expression of Erasp is regulated by Wg/Wnt1 under conditions of Rh-1^P37H^ misexpression-induced ER stress, the activity of Erasp appears to be modulated via transcriptional activation. However, it is also possible that the activity of Erasp is controlled by posttranslational modification, and this possibility warrants further investigation. Our immunoprecipitation assays suggest that Erasp physically interacts with DIAP1 (Fig. 5c). Through a GST pulldown assay, we were able to identify the binding domains in the two proteins, Erasp and DIAP1. We found that all three domains in DIAP1, namely, the BIR1, BIR2, and RING domains, were required for the interaction between the two proteins and for DIAP1 protein degradation (Fig. 5h). In addition, mutation of the putative residue that confers serine protease activity to Erasp, namely, serine-228, weakened the ability of Erasp to degrade DIAP1 (Fig. 5i). Taken together, our findings suggest that Erasp and *Drosophila* OMI/HTRA2 are involved in regulating apoptosis, although the mechanism underlying the activation processes of these proteins is quite different.

Our study demonstrates that the knockdown of Wg/Wnt1 delays the course of the retinal degeneration phenotype in the *Drosophila* model of ADRP. A number of *Drosophila* Rh-1 allele mutants, widely known as *ninaE*, serve as faithful models of human ADRP. These endogenous mutants have similar, if not identical, mutations to those found in humans, and they trigger retinal degeneration in an age-dependent manner^28,46^. Since the cause and pathological outcome of harboring rhodopsin mutations are similar between *Drosophila* and humans, it appears that the intermediate signaling responses are also conserved between the two species. Similarly, previous studies have shown that aberrant activation of Wnt signaling plays pathological roles in retinal diseases, including diabetic retinopathy and age-related macular degeneration^61–63^. As Wnt proteins also play a central role in various stages of retinal development, we speculate that Wnt signaling may play different roles depending on its environments, such as the developmental stage or cell type.

Similarly, we need to distinguish the apoptotic function of Wnt signaling between apoptosis that is programmed during development and apoptosis that results from ER stress. Wnt is considered to act as a morphogen, which means that its activity is concentration-dependent^64^. Although Wnt signaling is initiated by the binding of Wnt to its receptor, fz, many extracellular inhibitory components of Wnt signaling, namely, Dickkopf (Dkk), Soluble Frizzled-Related Proteins (SFRPs), and Wnt inhibitory factor (WIF), tightly regulate the binding process via physical interactions or modulation of binding affinity. These extracellular Wnt inhibitors are also known to activate signaling by regulating Wnt stabilization, secretion, or transport, depending on the physiological context^18^. Moreover, ectopic differences in nuclear components or intracellular components in certain cell types may affect Wnt signaling. Considering all the possible mechanisms by which Wnt signaling is regulated, changes in Wnt levels in cells or tissues that are affected by chronic or extreme ER stress can activate uncontrolled Wnt signaling and ultimately trigger apoptosis via the expression of pro-apoptotic proteins, such as Erasp. To summarize, we suggest that ectopic increases in Wnt expression via the recruitment of different types of Wnt regulatory proteins in certain cells suffering from extreme (chronic) ER stress lead to the activation of pathological Wnt signaling, which may differ from Wnt signaling during normal development. Again, since the p53 overexpression-induced cell death phenotype in the eye does not involve Wg/Wnt1 (Supplementary Fig. 4d,e), we propose that the role of Wg/Wnt1 in cell death is not general but more specific to certain stress-related signaling pathways. Altogether, our study highlights the potential for manipulating Wnt signaling to develop new approaches to therapeutic interventions in ER stress-related diseases.

## Supplementary information

Supplementary Information

Supplementary Table 1

Supplementary Table 2

Supplementary Table 3

Supplementary Table 4
