# Supplementary material for: Wg/Wnt1 and Erasp link ER stress to proapoptotic signaling in an autosomal dominant retinitis pigmentosa model
Source: Exp Mol Med. 2023 Jul 18;55(7):1544–55. doi: 10.1038/s12276-023-01044-7 (PMC10394004; doi:10.1038/s12276-023-01044-7)
Supplement: Supplementary file 1 — Supplementary Information [file 12276_2023_1044_MOESM1_ESM.pdf]

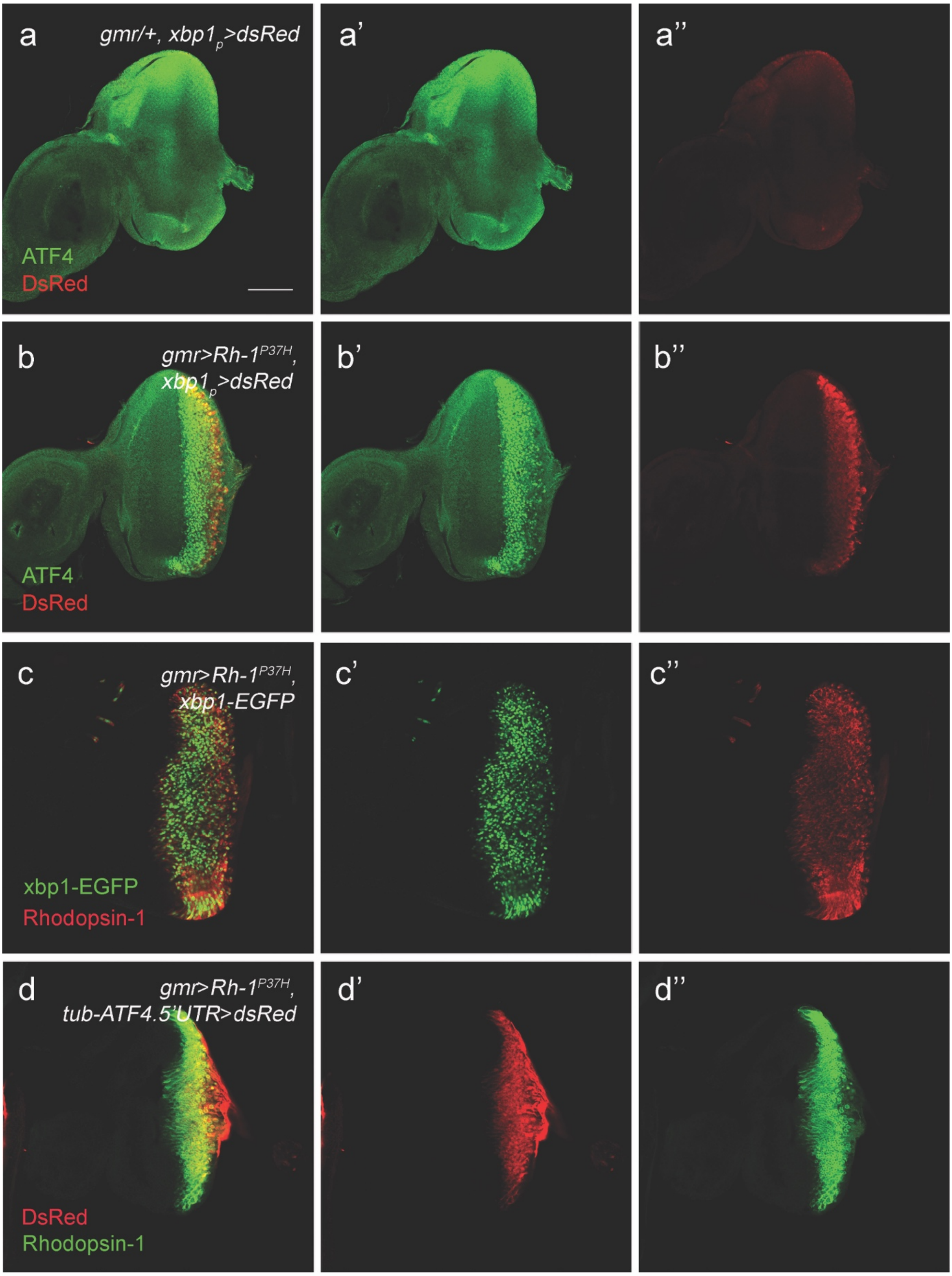


Supplementary Figure 1

**Supplementary Figure 1. ER stress reporter activation upon misexpression of the Rh-$1^{P37H}$ allele, Related to Figure 1.** The Rh-$1^{P37H}$ gene was misexpressed in developing eyes using the *GMR-GAL4* driver. (a and b) Eye imaginal discs expressing the ER stress reporter $xbp1_p$>dsRed alone (a) or together with Rh-$1^{P37H}$ (b). ATF4 was induced by Rh-$1^{P37H}$ (b) but not the control (a). (c) XBP1-EGFP reporter activation. (d) tub-ATF4. 5'UTR>dsRed activation. The scale bar represents 100 μm (a).

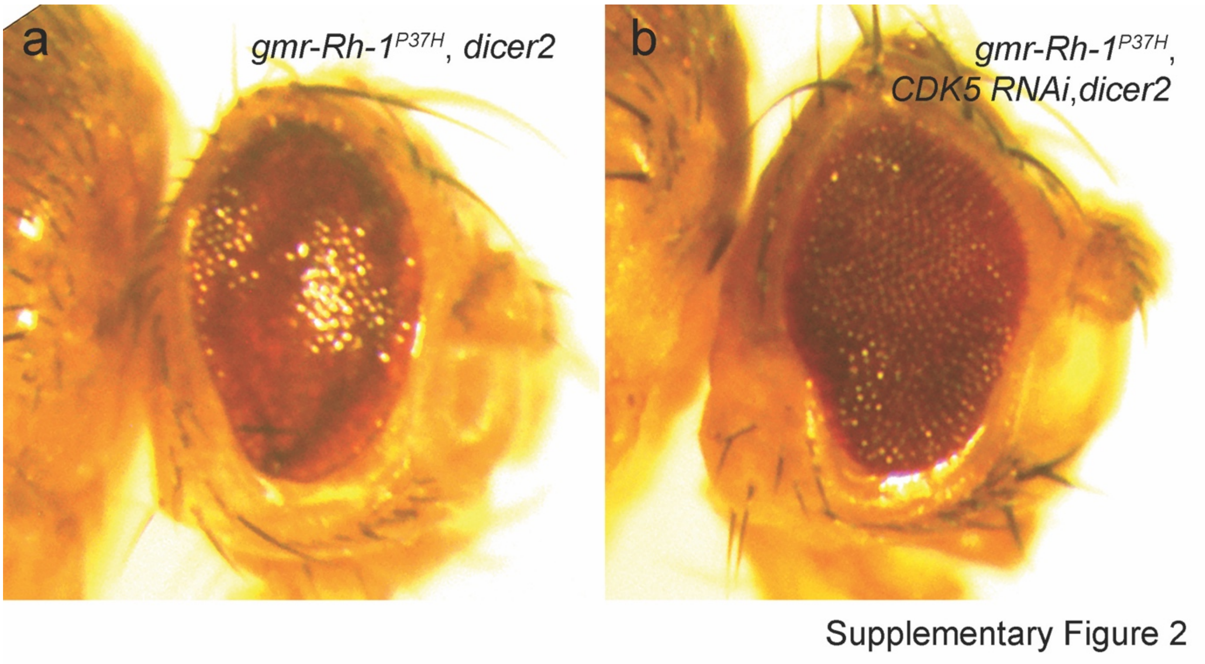


**Supplementary Figure 2. Eye phenotype observed on CDK5 knockdown, Related to Figure 1.** (a) Fly eye harboring *gmr-Rh-1*$^{P37H}$ and dicer2. (b) CDK5 knockdown fly eye in *gmr-Rh-1*$^{P37H}$-expressing flies.

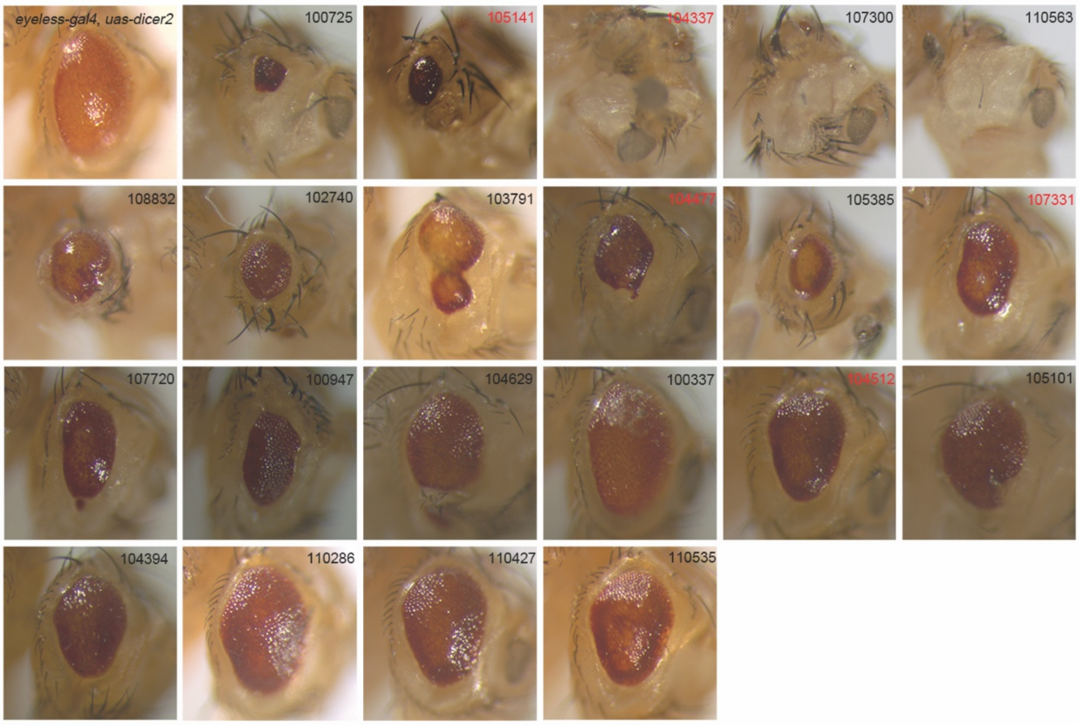


Supplementary Figure 3a

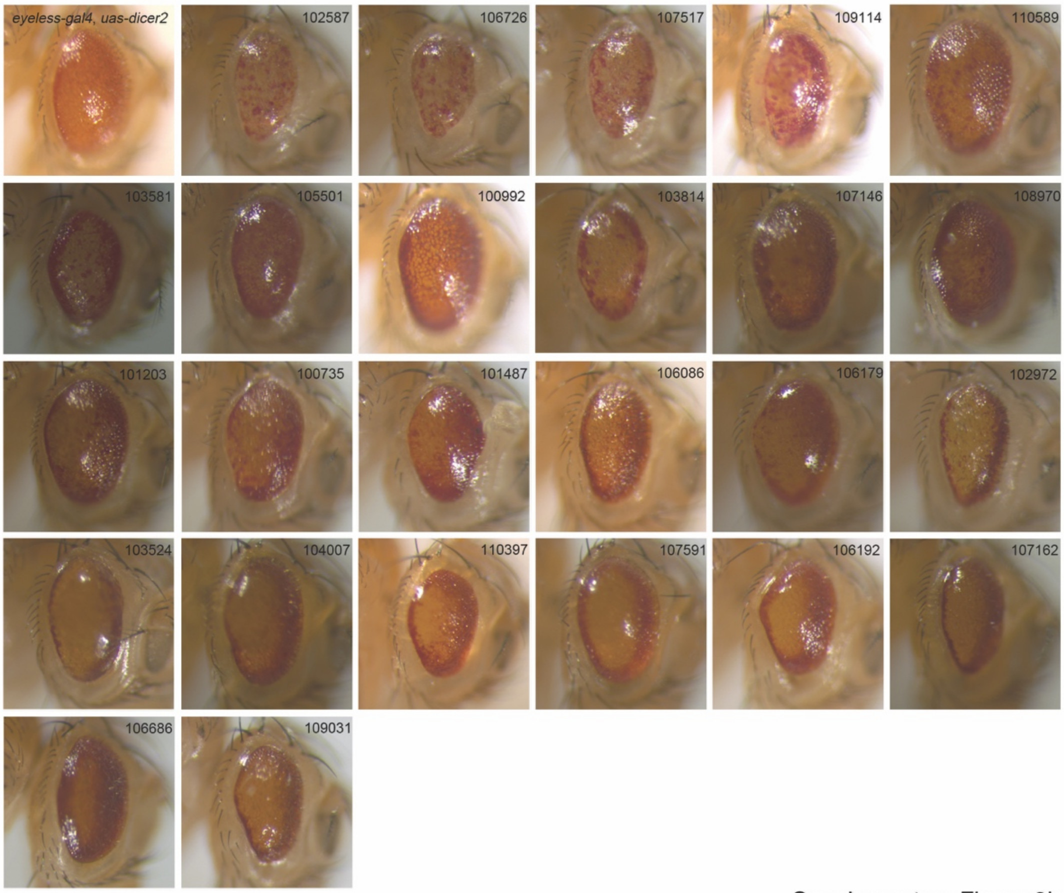


Supplementary Figure 3b

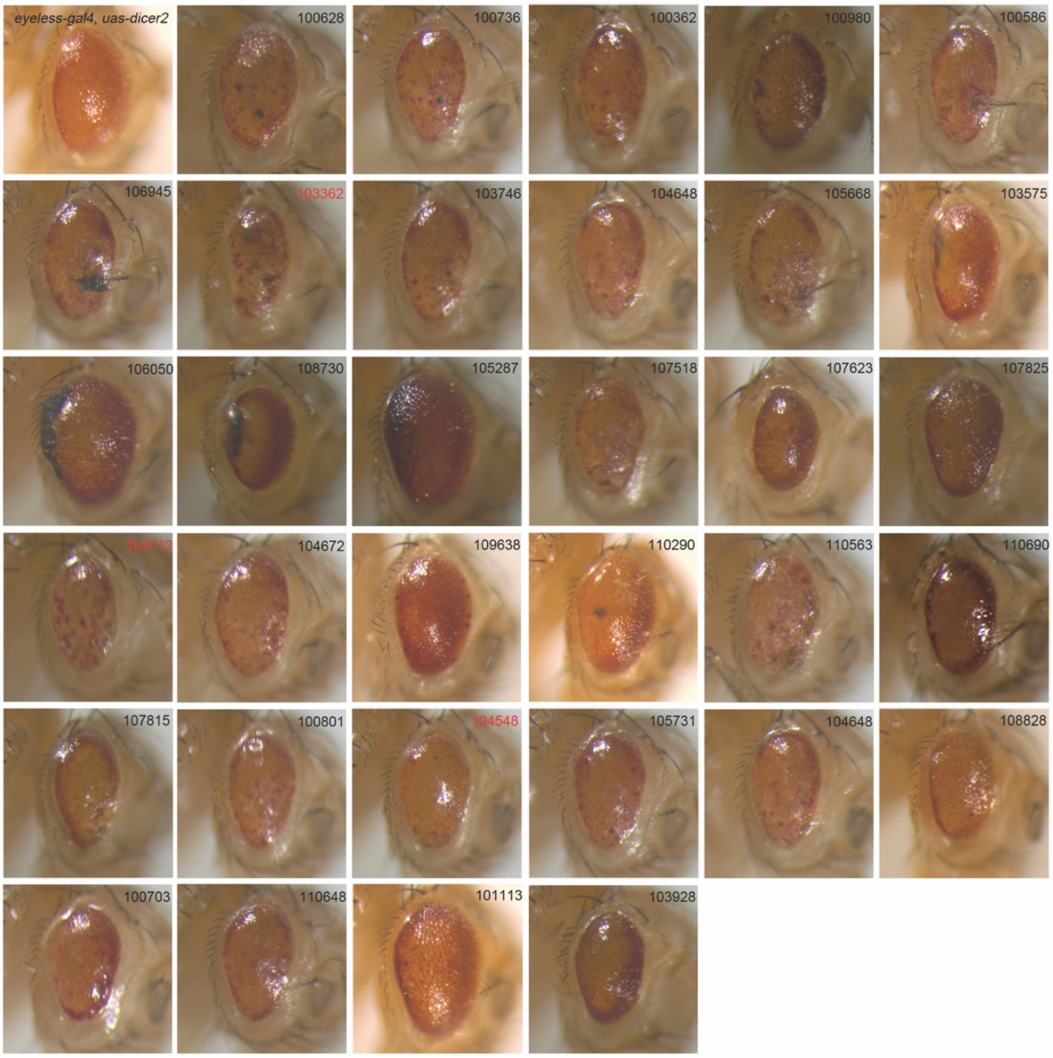


Supplementary Figure 3c

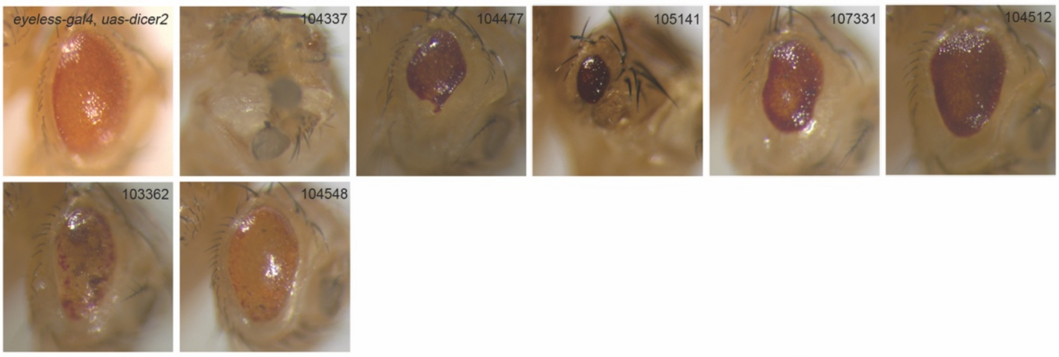


Supplementary Figure 3d

**Supplementary Figure 3. Eye phenotype observed on knockdown of gene in gmr-Rh-1$^{P37H}$ flies, Related to Figure 1.** The number indicates the VDRC number of the corresponding gene. (a) Knockdown of the indicated gene resulted in decreased eye size in

*gmr-Rh-1$^{P37H}$*-expressing flies compared to controls. The number in red font indicates the only knockdown effect caused by the indicated gene. (b) The images show the depigmented eye produced on gene knockdown in *gmr-Rh-1$^{P37H}$* expressing fly. (c) The *gmr-Rh-1$^{P37H}$*-expressing flies had black dots and eye scarring when the indicated gene was knocked down. (d) The eyes were altered only by knockdown of the indicated gene in wild-type flies.

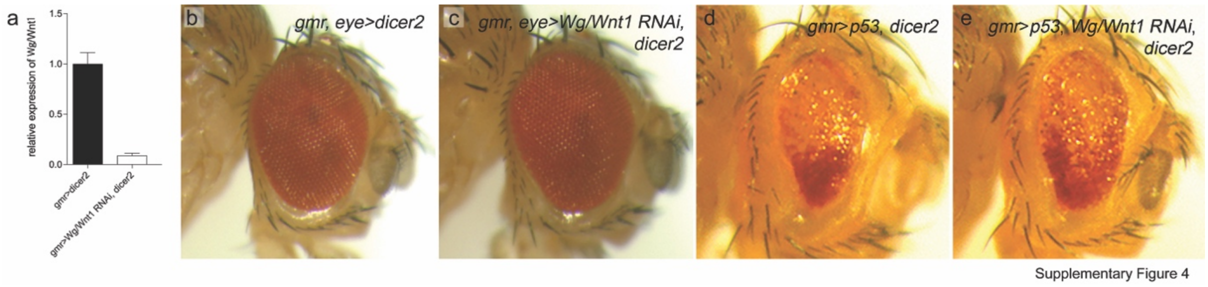


**Supplementary Figure 4. External adult eyes, Related to Figure 1.** (a-c) Eye phenotype of *Wg/Wnt1*-knocked down fly. (a) Knockdown efficiency of the Wg/Wnt1 RNAi line. (b) *DICER2*-expressing *Drosophila* eye (control). (c) *Wg/Wnt1*-knockdown *Drosophila* eye. (d-e) The eye phenotype of p53-expressing flies. (d) p53-expressing flies. (e) Wg/Wnt1 knockdown does not affect the rough eye phenotype caused by p53 overexpression.

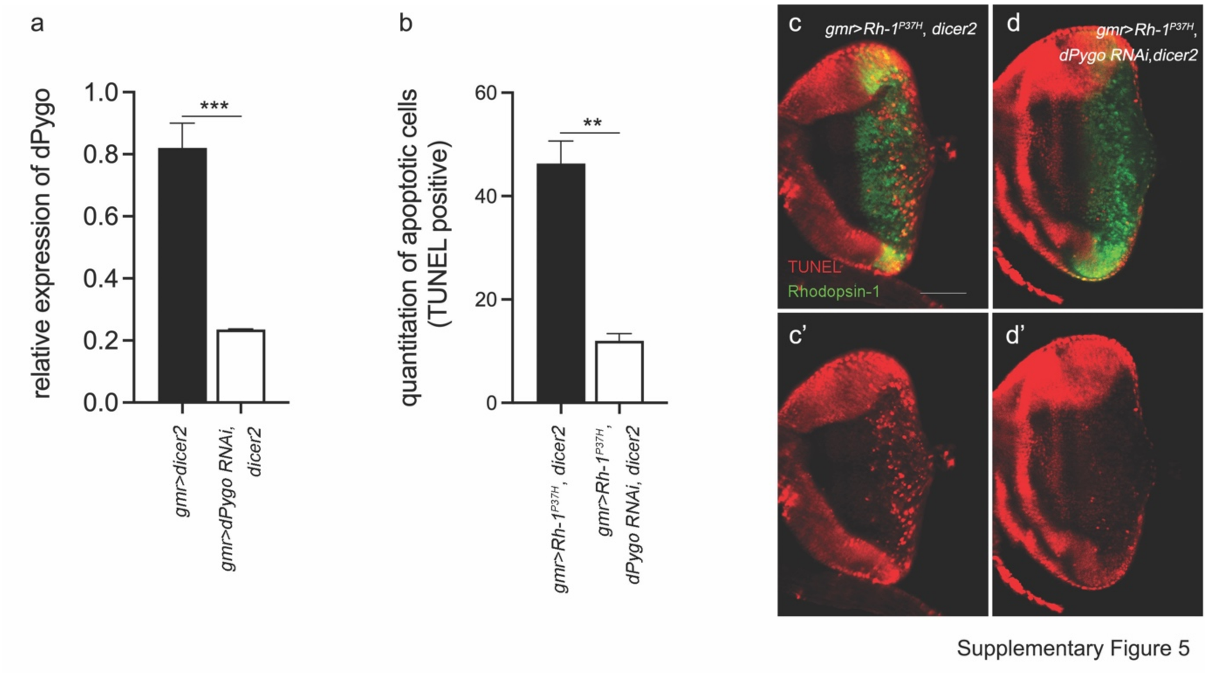


**Supplementary Figure 5. The knockdown of *dPygo* suppressed the Rh-1$^{P37H}$-induced apoptosis, Related to Figure 1.** (a) Knockdown efficiency of the *dPygo* RNAi line. (b) Comparison of the number of apoptotic cells between c and d. (c-d) Massive apoptosis caused by Rh-1$^{P37H}$ misexpression (c) was significantly suppressed in *dPygo* knocked down eye discs (d). *P* values were obtained using Student's *t*-tests. ***$P < 0.001$. The scale bars in c represent 50 μM.

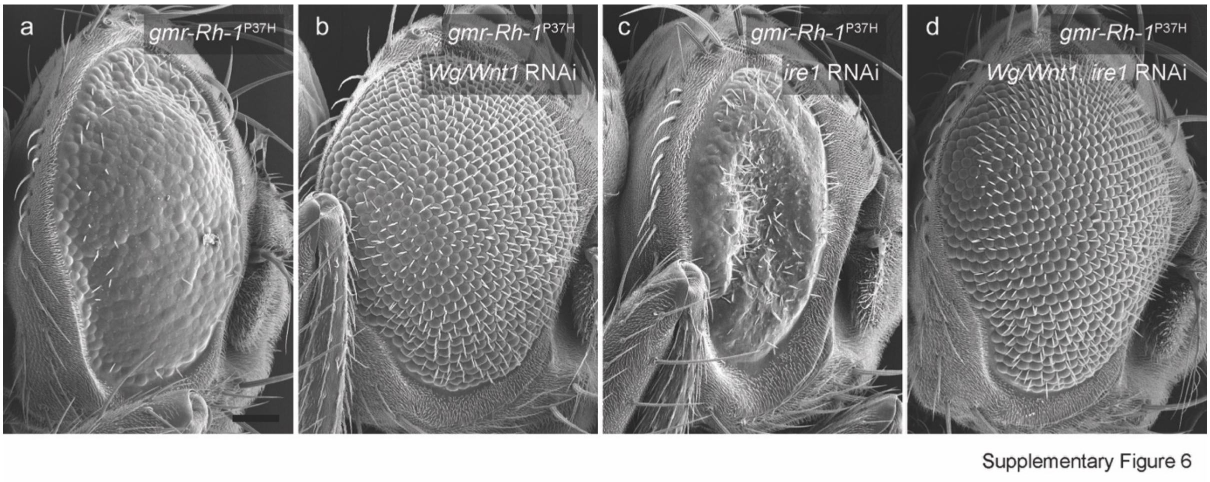


**Supplementary Figure 6. Enhanced Rh-1$^{P37H}$-induced apoptosis caused by *ire1* knockdown, Related to Figure 1.** (a) *gmr-Rh-1$^{P37H}$*-expressing flies. (b) *Wg/Wnt1* knockdown in *gmr-Rh-1$^{P37H}$*-expressing flies. (c) *ire1* knockdown in *gmr-Rh-1$^{P37H}$*-expressing flies. (d) Double knockdown of *Wg/Wnt1* and *ire1* in *gmr-Rh-1$^{P37H}$*-expressing flies.

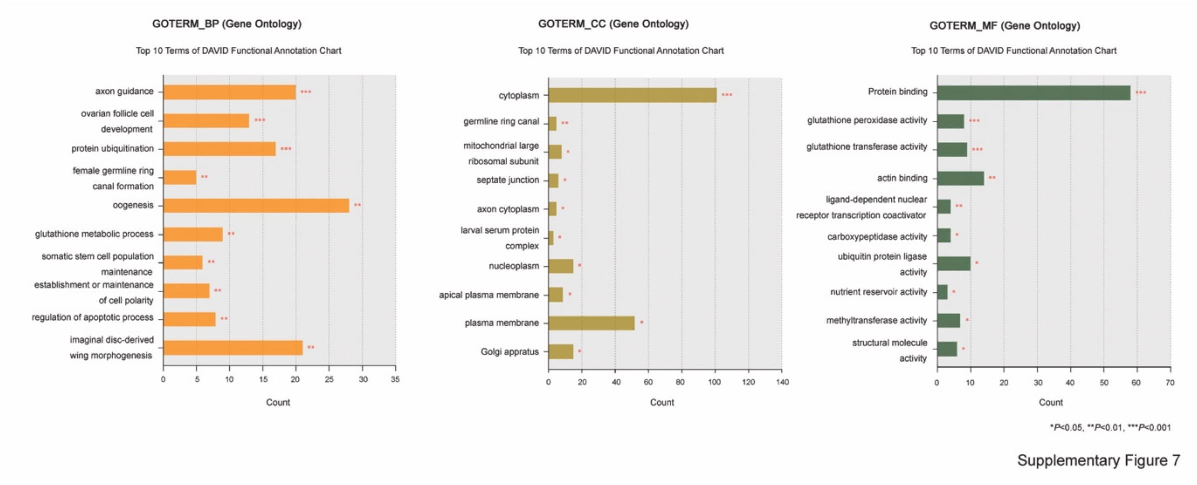


**Supplementary Figure 7. GO analysis of DEGs identified in RNA sequencing studies, Related to Figure 2**. Top 10 significantly enriched GO terms of the target genes in *GMR>DICER2 vs. GMR>Rh-1*$^{P37H}$, *DICER2*. Different colors represent the biological processes, cellular components, and molecular functions.

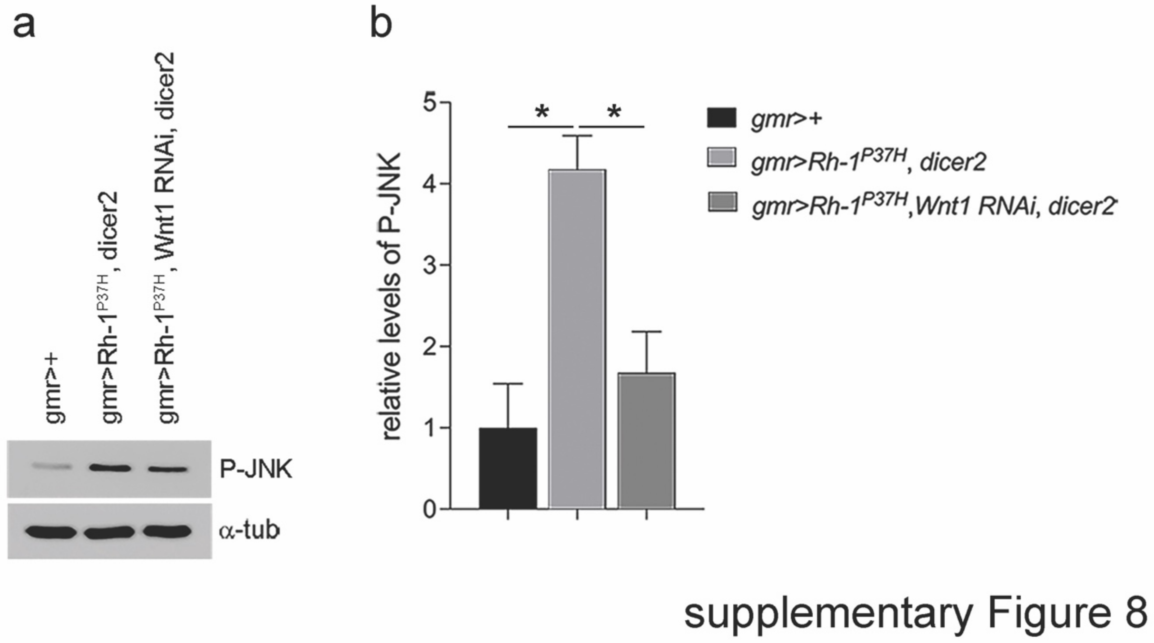


**Supplementary Figure 8. The JNK activation by misexpression of Rh-1$^{P37H}$ was mediated by noncanonical Wnt pathway, Related to Figure 1.** (a) The misexpression of Rh-1$^{P37H}$ in eye discs induces JNK phosphorylation, which is significantly reduced by Wg/Wnt1 knockdown. (b) Comparison of JNK activation, with the value from the control (*gmr*>+) set to 1.

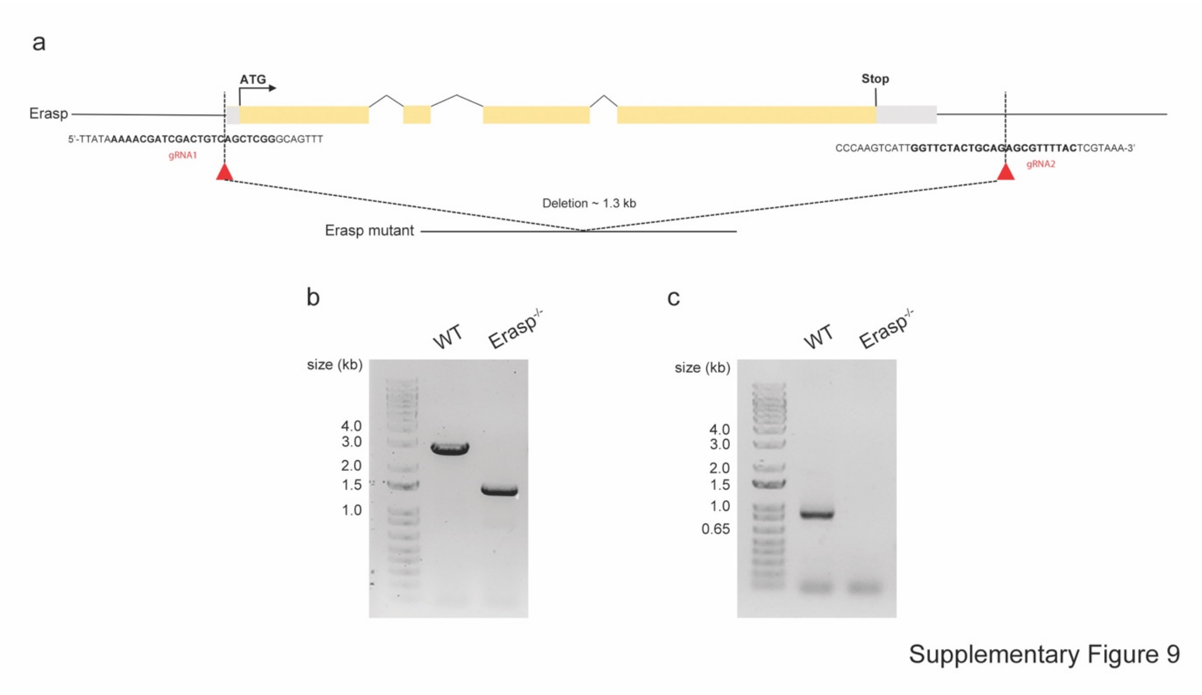


**Supplementary Figure 9. Generation of the Erasp mutant line, Related to Figure 3.** (a) The genomic loci of Erasp before and after deletion. The open reading frames and the cleavage sites used by gRNAs are indicated by yellow boxes and arrowheads, respectively. The gRNA-matching sequences are denoted in bold. The deleted region was confirmed by sequencing of the genomic DNA. (b) The deletion of Erasp in the genome was confirmed through genomic DNA PCR. (c) RT–PCR analysis confirmed the lack of *Erasp* transcripts in Erasp-/- flies.

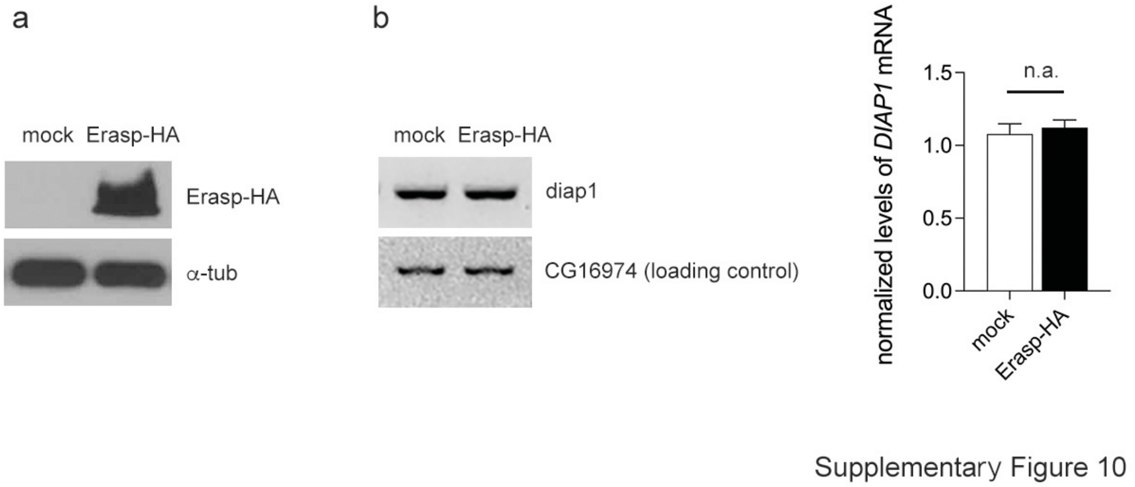


**Supplementary Figure 10. Expression levels of DIAP1 after Erasp overexpression, Related to Figure 5.** HA-tagged *Erasp* was transfected into *Drosophila* S2 cells (a). The *DIAP1* mRNA levels in these cells were measured by semiquantitative RT–PCR (b). Graphs indicate the quantification of *DIAP1* mRNA levels in the indicated S2 cells.

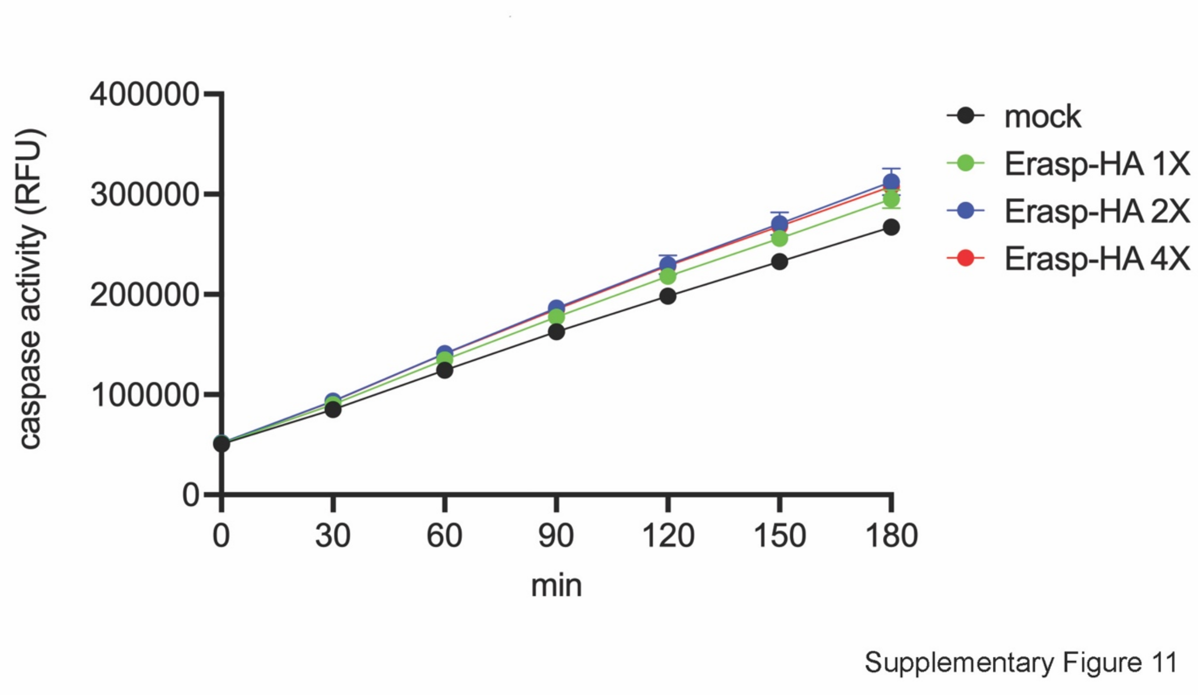


**Supplementary Figure 11. Caspase activity in *Drosophila* S2 cells after Erasp overexpression, Related to Figure 4**. Caspase activity was determined using a fluorogenic DEVD substrate. The measured activity was directly proportional to the amount of Erasp-HA.

**Supplementary Table 1. List of RNAi lines screened**

**Supplementary Table 2. Full list of transcripts that were differentially expressed in all samples**

**Supplementary Table 3. The analysis to identify the ER stress-related genes**

**Supplementary Table 4. The analysis to identify the transcriptional tagets of Wg/Wnt1 under ER stress**
